# Effects of Physiotherapy on Pain and Mouth Opening in Temporomandibular Disorders: An Umbrella and Mapping Systematic Review with Meta-Meta-Analysis

**DOI:** 10.3390/jcm12030788

**Published:** 2023-01-18

**Authors:** Manuel Arribas-Pascual, Sofia Hernández-Hernández, Christian Jiménez-Arranz, Mónica Grande-Alonso, Santiago Angulo-Díaz-Parreño, Roy La Touche, Alba Paris-Alemany

**Affiliations:** 1 Departamento de Fisioterapia, Centro Superior de Estudios Universitarios La Salle, Universidad Autónoma de Madrid, Aravaca, 28023 Madrid, Spain; 2 Motion in Brains Research Group, Centro Superior de Estudios Universitarios La Salle, Universidad Autónoma de Madrid, Aravaca, 28023 Madrid, Spain; 3 Instituto de Rehabilitación Funcional La Salle, Aravaca, 28023 Madrid, Spain; 4 Facultad de Medicina, Universidad CEU San Pablo, 28668 Madrid, Spain; 5 Instituto de Dolor Craneofacial y Neuromusculoesquelético (INDCRAN), 28023 Madrid, Spain

**Keywords:** temporomandibular disorders, manual therapy, therapeutic exercise, low-level laser therapy, meta-meta-analysis

## Abstract

The aim of this meta-meta-analysis was to assess the current evidence regarding the effect of physical therapy (PT) interventions on pain and functional variables in temporomandibular disorders (TMD). We conducted an umbrella systematic review (SR) and four meta-meta-analyses (MMA) and created an evidence map to determine the effectiveness of PT on pain intensity and maximum mouth opening in patients with TMD. The quality of the included SR was assessed with the AMSTAR 2, and the risk of bias with ROBIS. Of the 31 SR included in the umbrella SR, only 10 were included in the MMA. The MMA showed moderate effects for manual therapy and therapeutic exercise, and large effects for low-level laser therapy on improving pain intensity and maximum mouth opening in patients with TMD, with a limited to moderate quality of evidence. The overlapping analyses showed only a slight overlap for all the MMA according to the corrected covered area (range from 0.07 to 0.2), 23.1% to 41.6%. This umbrella SR showed that manual therapy and exercise interventions, as well as low-level laser therapy interventions, are effective in the reduction in pain intensity and improvement of maximum mouth opening in TMD. This article presents a synthesis of the available evidence related to the various physical therapy interventions used in patients presenting with temporomandibular disorders. These results could help clinicians to select the optimal intervention for their patients and to reject those that are less useful.

## 1. Introduction

Among the pathological conditions of the orofacial region, temporomandibular disorders (TMD) are among the most frequent once dental caries and periodontal diseases have been ruled out [1]. These represent a group of musculoskeletal disorders affecting the masticatory muscles, temporomandibular joints (TMJ), and related structures [2,3]; thus, they are classified as follows according to the Diagnostic Criteria for Temporomandibular Disorders (DC/TMD) Axis I, (1) pain-related disorders (including myofascial pain, myofascial pain with referral, local myalgia, arthralgia and headache attributed to TMD), and (2) TMJ intra-articular disorders (including disc displacement with or without reduction, degenerative joint disease and subluxation) [4]. The prevalence of TMD within the general population ranges from 10.8% to 55.9%, for which women (65.8%) between the age of 25 and 45 comprise the highest population [5,6,7]. TMD are characterized by orofacial pain as the main symptom, as well as limitation of mandibular movements [2,3]. Other clinical manifestations are muscle or joint tenderness on palpation, joint sounds and otologic complaints such as ear fullness, tinnitus and dizziness [2,3,8]. It has been described as an association between the presence of headache and TMD, the presence of headache among patients with TMD being more frequent [6,9]. Some authors also refer to the possible association with the cervical region, since it has been demonstrated that the treatment of this region could have a positive influence in patients with pain-related TMD [9]. However, the possible association between the TMD and the spinal posture is still a controversial topic [10].

Due to TMD complexity and multifactorial behavior, there is no single approach to treat these patients; thus, the literature suggests applying multimodal treatment, combining conservative therapies such as therapeutic exercise (TE), therapeutic education (TEd), cognitive-behavioral therapies, manual therapy (MT), and laser therapy, with invasive therapies such as dry needling (DN) [1]. Currently, conservative treatment has been recommended as the first choice for TMD [11,12,13]. A recent systematic review (SR) and meta-analysis suggests that among the conservative interventions, those that showed the most benefits for pain relief in patients with TMD are laser therapy and splint therapy [13]. However, a multimodal biopsychosocial behavior-based and multidisciplinary approach could be optimal [11,12,14]. To date, there is a significant body of evidence on the effects of various physical therapy (PT) interventions on the treatment of TMD, focused mainly on the effects of MT, TE, TEd, and electrotherapy on pain and functional variables; however, no compilation of all SR on the treatment of these patients has yet been performed.

Although it has been shown in various SR that a number of PT approaches are safe and simple interventions that could benefit patients with TMD, there remain controversies around the quality of evidence from these studies [15,16,17,18,19,20,21,22,23]. On the other hand, SR are usually published, and there is a lack of a compilation of all that information, which is why umbrella reviews are being carried out currently [24]. With umbrella reviews and meta-meta-analysis, we can perform a compilation of all the information of the different SR in order to achieve the most reliable evidence possible.

Due to the controversies around the effectiveness of PT treatment for patients with TMD, a joint assessment of the effects of various PT interventions through an umbrella SR and mapping the results to create a rapid and didactic view of the effects and target populations of these effects appears necessary to answer our research question: “What is the effect of PT interventions on the outcomes of patients with TMD?” The aim of this study was therefore to develop a mapping and umbrella review with a meta-meta-analysis (MMA) to synthesize and critically evaluate the current evidence for the effect of PT on TMD, specifically on pain and functional variables such as pain intensity (PI) and maximum mouth opening (MMO).

## 2. Materials and Methods

This study was conducted in accordance with the Preferred Reporting Items for Overviews of Systematic Reviews including the harm checklist, which consists of 27 items (56 sub-items), followed by a 5-stage process flow diagram (identification, screening, eligibility, inclusion, and separation of relevant studies) [25]. The protocol of this systematic review and meta-analysis was registered in PROSPERO (CRD42021234520).

### 2.1. Review Inclusion Criteria

The inclusion criteria we employed were based on the PICOS method, which regards the 5 essential elements of the research question in evidence-based practice: population, intervention, control, outcomes, and study design [25,26,27].

#### 2.1.1. Population

The participants selected for the articles were adults (18 years and older older) diagnosed by any type of health professional (doctor, dentist, and physical therapist) with any type of TMD including myogenous and arthrogenous TMD according to DC/TMD or RDC/TMD [4,28].

#### 2.1.2. Intervention and Control

Any type of physiotherapeutic intervention performed in isolation or combined with other treatment techniques was accepted. Control group interventions could be placebo, standard care treatment, or any other type of physiotherapeutic intervention.

#### 2.1.3. Outcomes

The outcomes employed to assess the results and effects of the various physiotherapeutic techniques in TMD were PI and MMO.

#### 2.1.4. Study Design

We selected systematic reviews with or without a meta-analysis of randomized controlled trials (RCTs), controlled clinical trials, or a combination of both, given that they are primary studies from which useful information can be extracted. There were no restrictions for any specific language as recommended by the international criteria [29].

### 2.2. Search Strategy

The SR search was conducted on 5 databases—PubMed, PEDro, Scielo, and LILACS—from inception to 26 April 2021; and on EBSCOhost from inception to January 2021. The last search was conducted on 26 April 2021. The search strategy combined medical subject headings (MeSH) and non-MeSH terms, adding Boolean operators (AND and OR) to combine them, and “systematic review” and “meta-analysis” were the filters added in the various databases when possible.

Keywords were as follows: “physical therapy”, “manual therapy”, “exercise”, “dry needling”, “soft tissue therapy”, “soft tissue mobilization”, “massage”, “massage therapy”, “therapeutic exercise”, “education”, “laser therapy”, “oral myofunctional therapy”, “relaxation”, “relaxation techniques”, “biofeedback”, “cold”, “thermotherapy”, “cryotherapy”, “heat”, “TENS”, “cold therapy”, “heat therapy”, “laser”, “electrotherapy”, “ultrasound therapy”, “microwave”, “temporomandibular joint”, “temporomandibular disorders”, “chronic temporomandibular disorders”, “temporomandibular disorder”, “myofascial masticatory pain”, “masticatory muscle pain”, “masticatory pain”, “headache attributed to TMD”, “headache attributed to temporomandibular disorder”, “disc displacement with reduction”, “temporomandibular degenerative joint disease”, and “temporomandibular subluxation”.

Terms and Boolean operators were adapted to the requirements of each database when necessary. We searched on a source of grey literature, such as Google Scholar, by hand-searching from inception to January 2021. We searched by reference checks of the SR included, we contacted an expert on the topic, and we manually searched in PubMed for SR according to various specific types of physiotherapy interventions. The search process was conducted by 3 independent reviewers (M.A., S.H. and C.J.) using the same methodology. To develop the search strategy, they were helped by an experienced researcher and expert on the topic (A.P.). Differences that emerged during this phase were resolved by consensus. The screening of the articles was performed by hand-searching, and we contacted authors by email if the full text was not available.

Appendix, Table A1 shows the search strategies for each database.

### 2.3. Selection Criteria and Data Extraction

In the first phase, 3 independent reviewers (M.A., S.H. and C.J.) performed a data analysis, assessing the relevance of the reviews regarding the study question, the objectives, and the inclusion and exclusion criteria. The analysis was performed based on information from each study’s title, abstract, and keywords. If there was no consensus or if the abstract did not contain enough information, the full text was reviewed.

In the second phase, the full text was reviewed to assess whether the studies met all the inclusion criteria. Differences between reviewers were resolved by discussion, and consensus was moderated by a fourth reviewer (A.P.). Data described in Section 3 were extracted by means of a structured protocol that ensured that the most relevant information was obtained from each study [30].

To structure the information extracted from each SR, 2 different data extraction tables were planned, the first dedicated to general SR information (number of studies included, study population, intervention performed, meta-analysis variables, and study results), and the second dedicated to the interventions studied (type of intervention, description, dosage, and follow-up time points). The specific inclusion criteria followed at each of the phases of the umbrella SR are those described in Section 2.1.

### 2.4. Methodological Quality Assessment

Two independent reviewers (M.A. and A.P.) assessed the methodological quality of the SR (with or without meta-analysis), assessing each of the studies based on the Modified Quality Assessment Scale for Systematic Reviews (AMSTAR 2) [31]. This approach has proven to be a valid and reliable tool for assessing the methodological quality of SR, comprising 16 domains with the simple answer options “yes” or “no”, and “partial yes” when there was partial adherence to the standard. Each answer adds 0 (no), 1 (partial yes), or 2 (yes) points to the total score, which can range from 0 to 32 points. Seven domains are considered critical, and 4 confidence levels emerge from their assessment: “high”, “moderate”, “low”, and “critically low” [31].

We calculated the kappa coefficient (k) and percentage agreement scores to assess reliability prior to any consensus and estimated the inter-rater reliability using k: (1) k > 0.7 indicates a high level of agreement between reviewers; (2) k 0.5–0.7 indicates a moderate level of agreement; and (3) k < 0.5 indicates a low level of agreement [32]. Disagreements on the final quality assessment score were resolved by consensus and, if necessary, by a third reviewer.

### 2.5. Risk of Bias Assessment

We assessed the risk of bias with the Risk of Bias in Systematic Reviews (ROBIS) tool [33], which consists of 3 phases: (1) relevance assessment (optional); (2) identification of concerns with the review process through 4 domains related to the study eligibility criteria: identification and selection of studies, data collection, study appraisal, and synthesis and findings; and (3) judgement on the risk of bias. The ROBIS tool includes signaling questions to evaluate specific domains to help judge the systematic review’s risk of bias, which could be answered as “yes”, “probably yes”, “probably no”, “no”, or “no information”. The risk of bias is therefore judged as “low”, “high”, or “unclear” [33].

Two independent reviewers (M.A. and A.P.) evaluated the risk of bias in the selected studies. Disagreements were resolved through consensus and, if necessary, by a third reviewer. We estimated the inter-rater reliability using the same kappa coefficient cutoffs described in the previous section.

### 2.6. Evidence Map

We created a visual map of the scientific evidence for each SR with meta-analysis to visually display the information as a bubble plot. The review information is based on 4 dimensions [34]:

1. Number of studies (bubble size): The size of each bubble is directly proportional to the number of original studies included in each of the SR.
2. Study population, intervention, and outcome variable (bubble aspect): The study population determined each bubble’s color. The outcome variable was represented by a different background within each bubble of each study (smooth background for PI and chequered background for MMO). The study intervention is represented by the symbol inside each bubble: “X” for combined MT and TE, and “-” for low-level laser therapy (LLLT).
3. Effect size (x-axis): Each of the reviews was classified according to the effect size. When the effect size showed benefits for the intervention group, the intervention was classified as “trivial” (standard mean difference [SMD] between 0.0 and 0.2); “small” (SMD between 0.2 and 0.6); “moderate” (SMD between 0.2 and 1.2), “large” (SMD between 0.0 and 0.2); “very large” (SMD between 2.0 and 4.0); and “extremely large” (SMD more than 4.0). When the effect of the intervention was not superior to the control, it was classified as “no significant difference”.
4. Strength of evidence (y-axis): The reviews were sorted into the following 4 categories according to the 2018 Physical Activity Guidelines Advisory Committee Grading Criteria (PAGAC) approach [35]: strong strength of evidence, moderate strength of evidence, limited strength of evidence, or strength of evidence unable to be assigned.

### 2.7. Qualitative Analysis

As described above, we relied on the assessments of each SR (with or without MA) for the methodological quality of the primary studies, using the AMSTAR for the included reviews and the PAGAC for assessing the evidence across SR. For the PAGAC analysis, the team assessed the strength of evidence based on the description of the findings provided in the studies and discussed as a group how to reconcile it according to Miake-Lye et al. [34].

### 2.8. Data Synthesis and Analysis

We performed the statistical analysis using meta-analyses with interactive explanations (MIX, version 1.7) [36]. We employed the same inclusion criteria for the review, adding 2 criteria: (1) results contained detailed information on the comparative statistical data (mean, standard deviation, and/or 95% confidence interval [CI]) of the main variables; and (2) data for the analyzed variables were represented in at least 2 studies.

We present the summary statistics in the form of forest plots [37], which consist of a weighted compilation of all SMDs and corresponding 95% CIs reported by each study, and provide an indication of heterogeneity among the studies. We examined the statistical significance of the pooled SMDs using Hedges’ *g* to account for a possible overestimation of the true population effect size in small studies [38]. The magnitude of *g* was interpreted according to a 4-point scale: (1) <0.20, negligible effect; (2) 0.20 to 0.49, small effect; (3) 0.50 to 0.79, moderate effect; and (4) ≥0.80, large effect [39]. We estimated the degree of heterogeneity among the studies by employing Cochran’s Q statistic test (*p* < 0.1 was considered significant) and the inconsistency index (I^2^) [40]. An I^2^ > 25% is considered to represent low heterogeneity, while an I^2^ > 50% is considered medium, and an I^2^ > 75% is considered to represent large heterogeneity [41]. The I^2^ index is complementary to the Q test, although it has a similar problem with power as the Q test with a small number of studies [41].

A study was therefore considered heterogeneous when it fulfilled one or both of the following conditions: (1) the Q-test was significant (*p* < 0.1); and (2) the result of I^2^ was >75%. To obtain a pooled estimate of the effect in the meta-analysis of the heterogeneous studies, we employed a random-effects model [42]. In addition, we decided to perform an analysis of the overlaps in the primary studies included in the final MMA, to assess the possibility that the data obtained for the effects of the interventions were over- or underestimated.

We analyzed the overlap of primary trials included at each of the meta-meta-analysis. Two methods were used, on one hand the percentage of overlapping (% overlap) was calculated as number of duplicated trials divided by the number of include trials. On the other hand, as recommended by Pieper et al., we calculated the “corrected covered area” (CCA) as a measure of overlapping: CAA = (N − r)/(r*c − r), where “N” is the number of included publications including double counting of overlapped trials, “r” is the number of included trials, “c” is the number of meta-analyses. The interpretation of CCA used to interpret the degree of overlap was 0–5 slight, 6–10 moderate, 11–15 high, >15 very high. We included a citation matrix to graphically represent the overlap [43]. We have also added a sensitivity analysis to include one more overlapping contrast as recommended by some authors [44].

## 3. Results

**Figure 1:**
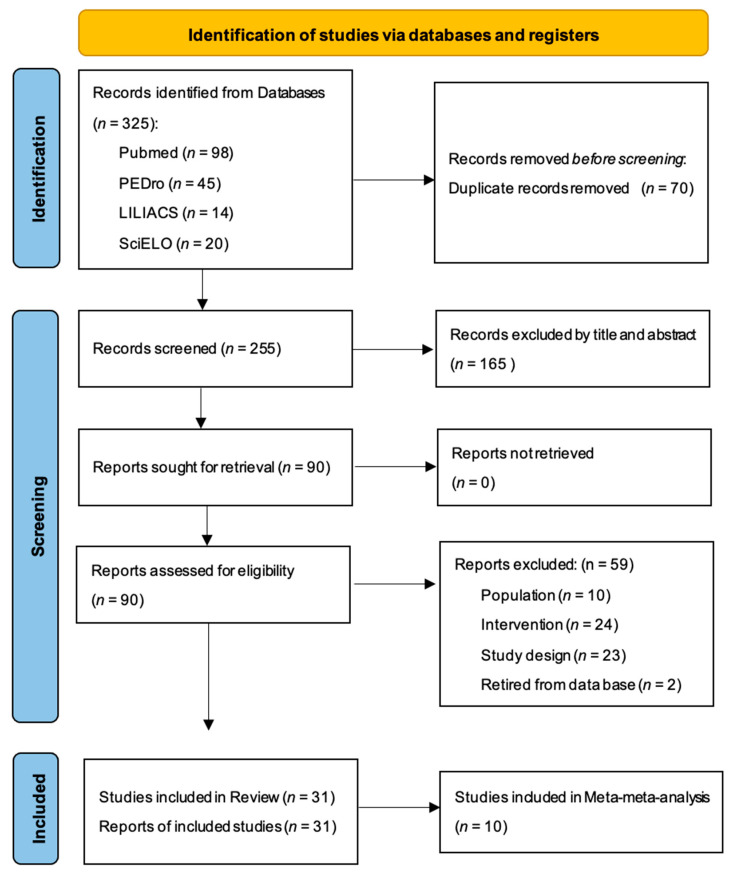
Flow chart of the research screening process.

The research screening process is shown in the form of a flow chart (Figure 1). We initially found 324 articles, with 255 remaining after removing duplicates. Following title and abstract screening, we selected 90 full-text review articles for further evaluation. Ultimately, 31 SR met the inclusion criteria. The Appendix, Table A2 shows the reasons for exclusion of each article in the full-text screening process. The publication dates of the included SR ranged from 2006 to 2021.

Fifteen of the included studies were SR [16,17,20,21,22,45,46,47,48,49,50,51,52,53,54], whereas the other sixteen were SR and meta-analyses [15,18,19,55,56,57,58,59,60,61,62,63,64,65,66,67]. Table 1 list the characteristics of the included articles, and Table 2 list the characteristics of the interventions analyzed in each SR.

After the data extraction, we could only conduct 4 independent MMA, which included 10 of the 31 SR: (1) a meta-analysis including 4 studies regarding MT and TE on PI [15,57,60,63]; (2) a meta-analysis including 3 studies regarding MT and TE on MMO [19,61,63]; (3) a meta-analysis including 4 studies regarding LLLT on PI [18,62,64,67]; and (4) a meta-analysis including 4 studies regarding LLLT on MMO [18,62,64,67].

### 3.1. Characteristics of the Included Systematic Reviews

Our umbrella SR included 31 SR (with or without MA), amounting to 463 original studies, 446 RCTs, and 17 non-RCTs included in 3 SR [49,51,54], with a total of 17,611 participants.

In terms of the population in the SR, all of them included patients with various diagnoses of TMD: 8 SR (*n* = 4286) included myalgia TMD [16,21,46,55,58,60,62,66]; 3 SR (*n* = 684) included intra-articular TMD [47,56,57]; 17 SR (*n* = 11,571) included any type of TMD [15,17,18,19,22,45,46,48,49,50,52,54,59,61,63,64,67]; and 2 SR (*n* = 850); 2 SR (*n* = 643) included headache attributed to TMD [58,65] and 2 SR (*n* = 850) included mixed TMD [20,53].

Referring to the diagnostic criteria used in the primary studies of the included SR, 18 (57%) SR used the research DC/TMD or the DC/TMD criteria [15,16,21,45,46,47,48,49,50,51,55,56,59,60,62,63,65,66]. In the remaining 13 SR, the diagnosis was made with non-standardized criteria based on signs and symptoms, such as the Helkimo index or Fonseca’s questionnaire, or it was not described [15,17,18,19,22,52,53,54,57,58,61,64,67].

In all the SR, there was at least one PT intervention group in the analysis of the primary studies. In nine SR (*n* = 5304) the intervention group used MT, either passive or active techniques focused on the TMJ or masticatory muscles, associated or not with other MT techniques such as cervical MT, or associated or not with other interventions such as TE [15,17,19,20,46,47,57,60,61]. In nine SR (*n* = 5195), the intervention group used TE, focused mainly on the TMJ, masticatory muscles, and postural correction, and associated or not with MT [15,19,20,22,46,47,51,58,60]. In three SR (*n* = 1570), the intervention group used DN, mainly for myofascial TMD [21,55,66]. In eight SR (*n* = 5510), the intervention group used LLLT, mainly comparing it with control groups and between various laser applications [18,52,53,54,59,62,64,67]. Only in one SR (*n* = 153) did the intervention group use biofeedback training [45]. In seven SR (*n* = 4302), the intervention group included various PT interventions, and the analysis was performed focused on each intervention and the controls or comparisons between interventions [16,48,49,50,56,63,65].

**Table 1:** Characteristics of the included systematic reviews.

Study	Nº, Type of Studies (Subjects)	Objectives of the Systematic Review	Population (Age, Type of TMD, Diagnosis Criteria)	Intervention and Comparison Groups	Nº of Studies Includes in Meta-Analysis (*n* = Subjects)	Outcome Measures	Results, MA Data Used for Meta-Meta-Analysis and Authors’ Conclusions
Al-Moraissi et al., 2020 [55]	21 RCTs (*n* = 959)	To compare and rank the treatment outcome of DN, acupuncture or wet needling using different agents, versus active and passive placebo in patients with myofascial TMD.	Mean Age (y): NA Diagnosis: Myofascial TMD according to RDC/TMD and DC/TMD.	Intervention groups: DN, acupuncture, instrumental injections of BTX and intramuscular injection of local anesthetic, granisetron and PRP. Comparison groups: Active sham placebo and passive placebo.	Post-treatment PI at the short-term follow-up: 12 (*n* = NA) Post-treatment PI at the intermediate-term follow-up: 10 (*n* = NA) Post-treatment MMO: 11 (*n* = NA) Post-treatment PPT: 10 (*n* = NA)	Post-treatment PI: VAS, NPRS and NRS Post-treatment MMO (mm): Caliper PPT (kg/cm^2^/s): Algometer	There was no difference between any of the treatments in any of the comparisons on post-treatment PI in the intermediate term (from one month to 6 months). There was a significant improvement of MMO with a mean increase of 2.37 mm after DN therapy (MD = 2.37; CI: 0.66 to 4.07) versus active placebo with very low-quality evidence and there was no difference after DN (MD = 0.09; CI: −0.53 to 0.50) when compared to placebo in the intermediate term (from 2 weeks to 6 months), with very low-quality evidence. Authors’ conclusions: PRP was shown to be the most effective treatment for pain reduction in the short term, followed by LA, dry needling and granisetron. On the other hand, in the intermediate term, LA was shown to be most effective, followed by dry needling, granisetron and BTX.
Al-Moraissi et al., 2020 [56]	36 RCTs (*n* = NA)	To identify the best treatment for adult patients with articular TMD and rank their effectiveness in PI reduction and improvement of jaw function.	Mean Age (y): Adults (NA) Diagnosis: Articular TMD according to RDC/TMD, DC/TMD and Other clear clinical diagnosis including signs and symptoms of TMD.	Intervention groups: PT, other conservative treatment, IAI, arthrocentesis alone or with or without IAI, arthroscopy with or without IAI, open joint surgery. Comparison groups: Control and placebo.	Network MA of 36 RCTs Control/placebo vs. other treatment in overall post-treatment PI: NA (*n* = NA) Control/placebo vs. other treatment in overall post-treatment MMO: NA (*n* = NA)	PI: VAS and NRS MMO (mm): NA	The non-invasive procedures of PT, conservative therapy, placebo/control provided significantly lower-quality outcomes relative to pain and MMO than invasive treatments. Overall post-treatment PI in articular TMD showed that PT compared with placebo/control had not statistically significant differences with very low-quality evidence (SDM = −0.45 [–1.04, 0.14]) and not statistically significant differences with a moderate-quality evidence for conservative treatments compared with placebo/control (SDM = −0.35 [–0.99, 0.30]). Overall post-treatment MMO in articular TMD showed that the conservative treatments compared with placebo/control showed not statistically significant differences with a low-quality evidence (SDM: −0.23 [–0.69, 0.23]). Authors’ conclusions: In articular TMD, minimally invasive procedures were shown to be significantly more effective than conservative treatment for both PI reduction and improvement of MMO, on a short- (up to 5 months) and intermediate-term (6 months to 4 years). The present NMA supports the challenge for a paradigm shift in articular TMD towards minimally invasive procedures as first-line therapy for the short-term improvement in pain and MMO.
Alves et al., 2013 [57]	2 RCTs (*n* = 175)	To assess the efficacy of mandibular manipulation alone or in combination with other techniques in the treatment of acute and chronic DDWR.	Mean Age (y): 41.5 (18–65) Diagnosis: DDWR according to MRI diagnosis.	Intervention groups: Mandibular manipulation alone or with other conservative treatment. Comparison groups: Palliative care, no treatment, arthroscopic surgery, medical managing.	Conservative therapy vs. other treatments on pain reduction: 2 (158) Conservative therapy vs. other treatments on mandibular function: 2 (159)	Pain Reduction: PI (VAS), MPQ and SSI Mandibular Function: MFIQ, CMI and DAL score MMO (mm): NA	At 8 weeks follow-up, there were not statistically differences on MMO, PI and mandibular function for physical medicine vs. palliative care and physical medicine vs. controls. At 60 month follow-up, there were not statistically differences in pain outcomes or mandibular function for rehabilitation vs. medical management, rehabilitation vs. arthroscopic surgery and rehabilitation vs. arthroplasty. MA showed non-significant differences favored conservative therapy vs. other treatments regarding the improvement of PI [SMD, −0.27; 95% CI, −0.61 to 0.08; *p* = 0.13); heterogeneity (Tau^2^: 0.00: Chi^2^: 0.00, df = 1 (*p* = 0.97); I^2^ = 0%); test of overall effect: Z = 1.50 (*p* = 0.13)] and the mandibular function. Authors’ conclusions: Mandibular manipulation (MT) in association with other conservative therapies may be considered and is sometimes suggested as the first choice for the treatment of anterior DDWR of the TMJ, because it is minimally invasive and inexpensive, avoiding unnecessary surgical procedures. Because good-quality evidence is also lacking for the alternatives, the intervention can be pragmatically used as an initial therapy.
Armijo-Olivo et al., 2016 [15]	48 RCTs (*n* = 2749)	To summarize the evidence, evaluate the quality of RCTs and determine the magnitude of the effect of the effectiveness of ET and MT in TMD.	Mean Age (y): NA (>18) Diagnosis: TMD according to RDC/TMD or any clinical diagnosis involving signs and symptoms of TMD.	Intervention groups: Any type of MT or TE intervention alone or in combination with other therapies. Comparison groups: Standard care, placebo, and control.	MMO in postural training vs. control group in myogenous TMD: 2 (*n* = 100) PI in MT + TE vs. control at 4 weeks to 3 months in arthrogenous TMD: 5 (*n* = 213) MMO in general jaw exercises vs. other treatments in mixed TMD: 7 (*n* = 270)	PI: VAS, MPQ, Total pain Rating Index, 11-point graded chronic pain scale, NRS and VRS MMO (mm): NA PPT (kg/cm^2^/s): Algometer	MA showed that posture correction exercises increased MMO compared with control in myogenous TMD. A general jaw exercise program showed no significant difference in the MMO (MD = −0.25 mm [−2.08, 1.57]; Tau2 = 0.00; x2 = 2.36; df = 6 (*p* = 0.85); *I*2 = 0%; Z = 0.27 (*p* = 0.79)) compared with ST, global re-education posture, ST + counselling, or standard conservative care, in mixed TMD. PI at 4 weeks to 3 months was significantly reduced in subjects receiving MT combined with TE when compared with ST, self-care, or medications (SMD = 0.40 [0.13, 0.68];]; Tau2 = 0.00; x2 = 2.90; df = 4 (*p* = 0.58); *I*2 = 0%; Z = 2.86 (*p* = 0.004)). Authors’ conclusions: No high-quality evidence was found about the effectiveness of TE and MT for treatment of TMD. Postural and mandibular exercises are positive for treating muscular and articular TMD. MT in the cervical spine decreases pain and increases mandibular ROM in muscular TMD. Exercise is not superior to other treatments in mixed TMD. A general exercise program was effective compared to arthrocentesis/arthrography for the treatment of articular TMD, with conservative treatments as the first line of treatment.
Brochado et al., 2019 [16]	35 RCTs (*n* = 1596)	To evaluate the effectiveness of non-invasive therapies to decrease pain and improve movements in TMD patients.	Mean Age (y): NA Diagnosis: Muscular TMD according to RDC/TMD, Helkimo index, Fonseca questionnaire and other non-standardized evaluation protocols.	Intervention groups: Non-invasive therapies Comparison groups: Other therapies, placebo/sham, and no treatment.	No MA	PI: VAS (0–10) and Verbal Pain Scale (0–10) MMO (mm): Millimeter ruler and caliper Mandibular Function: NA	The results demonstrated that 27 (77%) of the studies presented reduction in PI and 22 (62%) improvement in mandibular function. When analyzing the differences between treatments at the end of the protocols, 20 (57%) of the studies showed no significant difference in PI. Significant improvement in MMO was described only in 13 (37%) studies using LLLT (17%), acupuncture (6%), MT (6%), TENS (3%), OMT (3%), and benzodiazepine (3%). The combination of MT and oral TE/behavioral education (9%) or LLLT and oral TE (3%) had significantly better results than the therapies alone or placebo. Authors’ conclusions: In this review, non-invasive therapies were the first choice for TMD patients and all of them improve, at least partially, TMD signs and symptoms. Therefore, non-invasive treatments can provide pain relief and should be prescribed before surgical procedures. LLLT, ST, oral TE/behavioral education were the most used therapies. LLLT was the therapy with the higher number of studies showing positive results compared to placebo, control, or other therapies
Calixtre et al., 2015 [17]	8 RCTs (*n* = 331)	To evaluate the isolated effect of MT in improving TMJ function.	Mean Age (y): NA (18–65) Diagnosis: TMD according to NA	Intervention groups: MT Comparison groups: Placebo, standard treatment, and other treatments.	No MA	PI: VAS MMO (mm): Caliper PPT of the masticatory muscles (kg/cm^2^/s): Algometer	Moderate and low evidence that myofascial release and massage techniques are more effective than placebo or no intervention for MMO and pain outcomes. There is also moderate evidence that no significant difference exists between myofascial release and toxin botulinum for improvement on the same outcomes. There is moderate evidence that atlanto-occipital joint thrust manipulation is more effective than placebo in improving MMO in individuals with TMD. However, there is low evidence that C7-T1 thrust manipulation does not improve PPT in subjects with TMD when compared to placebo manipulation. In addition, an upper cervical mobilization showed high-quality evidence on reducing PI and increasing PPT comparing to placebo. MT osteopathic technique protocols showed greater MMO, PI and PPT improvement when compared to a usual care. Authors’ conclusions: MT reduces PI and increases MMO and PPTs in TMD, depending on the technique used.
Chen et al., 2015 [18]	14 RCTs (*n* = 454)	To evaluate the effectiveness of LLLT for patients suffering from TMD.	Mean Age (y): NA Diagnosis: Muscular, articular, or mixed TMD according to NA	Intervention groups: LLLT Comparison groups: Placebo LLLT.	PI (VAS) at the final follow-up point: 10 (368) Change in PI (VAS) between baseline and end of the follow-up: 8 (255) Active MMO at the follow up time point: 5 (184) Passive MMO at the final follow-up time point: 2 (104)	PI: VAS TMJ function (mm): Active MMO, Passive MMO, PE and LE	MA showed that -significant differences between LLLT and placebo on PI at the final follow-up time point [WMD = −19.39; 95% CI = −40.80 to 2.03; heterogeneity (Tau^2^:1147.16: Chi^2^: 808.30, df = 9 (*p*< 0.00001); I^2^ = 99%); test of overall effect: Z = 1.77 (*p* = 0.08)] and on PI change between baseline and end of the follow-up. Significantly better active MMO in the LLLT group vs. placebo [WMD = 4.18; 95% CI = 0.73 to 7.63; heterogeneity (Tau^2^: 10.21: Chi^2^: 14.57, df = 4 (*p* = 0.006); I^2^ = 73%); test of overall effect: Z = 2.38 (*p* = 0.02)] and better passive MMO in the LLLT group at the final follow-up time point. Additionally, there were significantly better right LE in the LLLT group and significant better PE in the LLLT group, but no significant difference in left LE between groups at the final follow-up time point. Authors’ conclusions: This study indicates that using LLLT significantly improve the functional outcomes but has limited efficacy in reducing PI in patients with TMD.
de Melo et al., 2020 [46]	5 RCTs (*n* = 279)	To evaluate the effectiveness of MT in the treatment of myofascial TMD.	Mean Age (y): NA (12–69) Diagnosis: Myofascial TMD according to RDC/TMD	Intervention groups: MT only, MT with counselling Comparison groups: No intervention, BTX injection, home PT and counselling.	No MA	PI: NA Oral Function: NA	The effectiveness of MT was closely related to counselling techniques. Their effectiveness was noticeably higher when these two types of treatment were combined. MT was better than no treatment in one study and better than counselling in another study. However, MT combined with counselling was not statistically better than counselling alone, and MT was not more efficient than BTX. MT combined with home therapy was better than home therapy alone in one study. Authors’ conclusions: The frequent applications of botulinum toxin represent a high-cost treatment with statistically similar efficacy to MT. Although no superiority was found for MT, it is a low cost, reversible, non-invasive, and efficient treatment for myofascial TMD pain.
Dickerson et al., 2017 [19]	6 RCTs (*n* = 419)	The effectiveness of TE for individuals with TMD	Mean Age (y): NA (13–75) Diagnosis: TMD according to NA	Intervention groups: TE (mobility and mixed exercise therapy) Comparison groups: Placebo and other treatments.	TE vs. control in PI: 3 (*n* = 152) TE vs. control in function: 2 (*n* = 78) TE vs. control in TMJ ROM (MMO, LE and PE): 3 (*n* = 172)	PI: VAS, SF36 pain, and others NA Function: SF36, MFIQ, and others NA Temporomandibular joint ROM (caliper): MMO (mm), LE (mm) and PE (mm)	This review indicates TE had moderate treatment effects in the short-term and varying amounts of long-term treatment effects in patients with TMD. TE does not appear be a significant direct improvement with oral functional improvements despite improvements of ROM. The improvements of ROM came from the TE emphasizing mobility interventions and a mixed multidimensional treatment program. Although the mixed TE approach twice weekly appears to be the most effective treatment method for pain outcomes. MA showed thar TE was superior to control only in the mixed approach intervention in reducing PI (SMD = 0.824 with a 95% CI; *p* = 0.011). Statistically insignificant regarding function between intervention and control groups between all TE groups. A significant difference was demonstrated through assessment of MMO favoring the mixed approach and mobility groups (SMD = 0.820 with a 95% CI; *p* = 0.018). Authors’ conclusions: Patients with TMD have less PI and better jaw ROM after TE targeting mobility or a mixed, multidimensional approach.
Florjanski et al., 2019 [45]	10 RCTs (*n* = 153)	To determine the effects and efficiency of masticatory muscle activity management based on BF.	Mean Age (y): NA (20–60) Diagnosis: TMD according to RDC/TMD, American Academy of sleep medicine criteria, self-report sign and symptoms and electromyography monitoring by night.	Intervention groups: BF training (visual, audio, and vibratory) and CES Comparison groups: TENS, ST, and no intervention.	No MA	PI: VAS (0–10) and NRS (0–10) PPT (kg/cm^2^/s): Algometer Muscle activity: Electromyography (EMG) Sleep bruxism episodes (nº): EMG	The results showed a significant decrease in PI, reduction in EMG of masticatory muscles and a significant decrease in sleep bruxism events by using BF training but no changes in PI and PPT by using CES. Most of the studies suggested a significant correlation between usage of biofeedback and reduction in masticatory muscle activity, with a very low to moderate quality of evidence. Only in one study, the tendency was marked but not statistically significant with a low quality of evidence. Authors’ conclusions: BF is useful in decreasing masticatory muscle activity. However, further studies on a larger group of participants considering coexisting genetic and environmental factors that can modify the effect of biofeedback on masticatory muscles are needed to verify the results of the treatment and long-term follow-ups to clarify permanence. Additionally, the efficiency of different protocols remains unclear.
Fricton et al., 2009 [58]	10 RCTs (*n* = 423)	To examine the effectiveness of TE interventions in the management of TTH and jaw pain in myofascial TMD.	Mean Age (y): NA Diagnosis: Muscular TMD, headache attributed to TMD and TTH according to NA.	Intervention groups: TE and postural improvement Control groups: No treatment, placebo, and other treatments.	TE compared with no treatment or placebo (education) in PI: 4 (*n* = 159)	Headache and jaw PI: NA MMO (mm): NA	Jaw-stretching exercises, ice, stretching exercises, massage, and relaxation therapy reduce PI and frequency of headache in headache attributed to TMD or muscular TMD vs. no treatment. Posture exercises with palliative instructions improved head and neck PI significantly more than a placebo-like palliative instruction only. Addition of posture correction was not associate with a net benefit over that of CBT alone or self-management alone. Addition of a TE program to other interventions for PI on headache and myofascial TMD, including patient education, ST, massage, heat, and other PT modalities did not show differences between groups with or without the exercise program. The MA demonstrated that there is a small but statistically significant trend for a favorable effect of TE compared with no treatment or placebo in the reduction in PI in patients with muscular TMD. Authors’ conclusions: The results suggest that exercise, particularly stretching and postural relaxation, has therapeutic value for PI on TTH and myofascial TMD, and should be included in the treatment regimen for these conditions. Although this review provides support for the use of exercise in treating TTH and myofascial TMD, it is still unclear what exercises work best, on what patients they work best, what outcomes can be expected, and what factors cause treatment failure.
Herrera-Valencia et al., 2020 [20]	6 RCTs (*n* = 304)	To evaluate the medium- and long-term efficacy of MT for TMD, alone or in combination with TE.	Mean Age (y): 42.5 (34–51) Diagnosis: Mixed TMD: mouth opening pain and/or limitation, myofascial symptoms, non-reducing disc displacement.	Intervention groups: MT or MT + TE Comparison groups: TE, BTX injections, education, stand self-care.	No MA	PI: VAS Active MMO (mm): NA Passive MMO (mm): NA PPT: Algometer	A significant improvement in PI around (4/10 points) and MMO (4.35–15 mm) compared to baseline was observed after MT. Comparing the effects of MT, TE and education, the differences in PI and MMO seemed to be non-significant in the medium term (3–6 months). The reason why these differences in results are observed between the different studies at 3 months may be the types of TE used. Authors’ conclusions: MT seems to be an effective treatment for TMD in the medium term, although the effect appears to decrease over time. However, when complemented with TE, these effects can be maintained in the long term.
Jing et al., 2021 [59]	16 RTCs (*n* = 538)	To compare the effects of different energy density LLLT in the management of TMD patients.	Mean Age (y): NA (14–76) Diagnosis: TMD according to RDC/TMD, American Academy of Orofacial Pain criteria.	Intervention groups: Different LLLT applications (d1: <10 J/cm^2^, d2: 10–50 J/cm^2^, d3: 50–100 J/cm^2^ and d4: >100 J/cm^2^) Comparison group: Placebo LLLT and control.	LLLT (d1) vs. placebo at 1 month follow-up on PI reduction: 5 (*n* = 160)	PI: VAS (0–10)	The MA showed that when comparing LLLT vs. placebo at 1 month follow-up on PI reduction, overall effect of LLLT showed no statistically significant differences with a low quality of evidence. Only d1 LLLT (<10 J/cm^2^) showed more PI reduction than placebo but no statistically significant difference was found. There was no statistically significant difference on PI reduction in favoring placebo vs. d3 LLLT (> 50 J/cm^2^). Authors’ conclusions: When energy density is not greater than 10 J/cm^2^, LLLT makes statistically significant pain reduction in the initial management of TMD. One month later, LLLT is more effective than placebo in pain management, but the result does not reach the point of statistical significance. For clinical application, d1 (energy density no greater than 10 J/cm^2^) LLLT is recommended for short-term pain management of TMD patients.
La Touche et al., 2022 [47]	10 RCTs (*n* = 509)	To analyze the effectiveness of exercise and MT interventions in patients with disc displacement without reduction.	Mean Age (y): 43 (12–74) Diagnosis: Disc displacement without reduction according to DC/TMD with or without magnetic resonance imaging (MRI).	Intervention groups: TE or MT, in combination or in isolation Comparison groups: Other treatment or no treatment.	No MA	PI: VAS or NRS MMO (mm): Millimeter ruler and Micrometer caliper	Limited evidence exists to suggest that TE significantly improves MMO in patients with disc displacement without reduction compared to ST; however, results regarding PI reduction were similar for both treatments. Limited evidence also suggests that non-supervised and supervised exercise significantly improves PI after surgical treatment in patients with disc displacement without reduction. Authors’ conclusions: Results show that interventions based on TE, or MT may be beneficial and play a role in the treatment of disc displacement without reduction. Limited evidence suggests that exercise significantly improves MMO in comparison to ST. However, due to the heterogeneity of the included studies, these results should be interpreted with caution; it is not possible to determine which is the best type of intervention or which parameters might be most beneficial in these patients.
La Touche et al., 2020 [60]	6 RTCs (*n* = 163)	To evaluate the effectiveness of cervical MT in patients with TMD and to compare the effectiveness of cervical MT vs. cervical MT + craniomandibular MT treatment in patients with TMD.	Mean Age (y): NA (18–65) Diagnosis: Myofascial or mixed TMD according to RDC/TMD.	Intervention groups: Cervical MT and cervical MT + craniomandibular MT Comparison groups: Placebo, sham, no intervention, and other non-MT interventions.	Cervical MT vs. Other Non-MT interventions on PI: 3 (*n* = 130) Cervical MT vs. Other Non-MT interventions on PPT in temporalis muscle: 3 (*n* = 130) Cervical MT vs. Other Non-MT interventions on PPT in masseter muscle: 3 (*n* = 130) Cervical MT vs. Cervical MT + Craniomandibular MT on PI: 2 (*n* = 88) Cervical MT vs. Cervical MT + Craniomandibular MT on MMO: 2 (*n* = 88)	Pin: VAS (0–10) and NRS (0–10). PPT (kg/cm^2^): Algometer MMO (mm): NA	When comparing cervical MT vs. other non-MT interventions, it was observed significant improvements in PI levels and PPT in masseter and temporalis muscles (0.15 to 1.5 kg/cm ^2^) for the cervical MT interventions. When comparing cervical MT vs. cervical MT + Craniomandibular MT significant reduction was found in PI at 3- and 6-month follow-up for both interventions, but the combined intervention showed greater improvement than the cervical MT intervention at 6-moths follow-up, in one study. Improve in MMO was significantly higher with the combined intervention at 3-month follow-up. The MA showed that when comparing cervical MT vs. other non-MT interventions, statistically significant differences in short-term PI reduction with a large clinical effect (SMD = −1.49, 95% CI = −2.45 to 0.54, Q = 10.75, *p* = 0.05, I^2^ = 81.3%) with no evidence of publication bias Egger’s test (SE = 1.461, T = −5.331, *p* = 0.118). Statistically significant differences in short-term increases in masseter PPT with a large clinical effect and for short-term temporalis PPT with a moderate clinical effect, with no evidence of publication bias for the masseter studies but there was a bias for the temporalis studies. When comparing cervical MT vs. cervical MT + craniomandibular MT, there were statistically significant differences in the short-term reduction in PI with large clinical effects, and statistically significant differences for MMO in the short-term reduction in pain-free MMO with large clinical effect. Authors’ conclusions: Cervical MT is more effective in decreasing PI than placebo MT or minimal intervention, with moderate evidence and cervical MT + craniomandibular MT achieved greater short-term reductions in PI and increased MMO over cervical intervention alone in TMD and headache, with low-quality evidence. These conclusions should be interpreted with caution due to the scarce evidence found in the literature.
Machado et al., 2018 [21]	18 RCTs (*n* = 412)	To evaluate the effectiveness of the injection of different substances and DN in the treatment of myofascial TMD pain.	Mean Age (y): NA (18–69) Diagnosis: Myofascial TMD according to RDC/TMD, American Academy of Orofacial Pain criteria, classification of the International Headache Society and other non-standardized criteria.	Intervention groups: DN and wet needling Comparison groups: Wet needling, sham needling, and other treatment.	No MA	PI: VAS (0–10) and NRS (0–10). PPT (kg/cm^2^ or kPa or kg or kgf): Algometer MMO (mm): NA	When comparing DN vs. substance injection, there were favorable results for PI in all groups, but none of the studies showed statistical difference between them. When comparing DN vs. substance injection vs. sham needling vs. other treatments, lidocaine and laser therapy were all effective for deactivation of trigger points. When comparing DN vs. sham needling, DN was efficient in improving PPT in two studies and MMO in one study. When comparing ND vs. other treatments, there was a greater reduction in PI at rest and on masticatory function for DN in comparison with a methocarbamol/paracetamol combination, but no significant improvement in MMO was observed. Authors’ conclusions: Definitive conclusions about the therapies evaluated cannot be made due to the lack of adequate quality of the selected studies.
Martins et al., 2016 [61]	8 RCTs (*n* = 375)	To analyze the effectiveness of MT in TMD patients and compare them to control treatments.	Mean Age (y): NA (12–44) Diagnosis: TMD according to NA.	Intervention groups: Active and passive interventions of MT Comparison groups: Active control groups.	MT vs. control PI during active MMO: 3 (*n* = 128) MT vs. Control on active MMO: 5 (*n* = 184)	PI: VAS and NRS. TMJ ROM (mm): Active MMO, Passive MMO, Pain-free MMO, LE, PE	MA showed that there was a significant difference and a large effect in favor of MT when compared to active control on active MMO (SMD = 0.83 [0.42, 1.25]; Tau^2^ = 0.10; Chi^2^ = 7.19; df = 4 (*p* = 0.13); I^2^ = 44%; Z = 3.93 (*p* < 0.0001)) and significant difference and large effect in favor of MT when compared to active control group on PI during active MMO. Authors’ conclusions: Significant results for MT to increase active MMO and decrease PI during active MMO. Musculoskeletal manipulations approaches are effective for the treatment of TMD. In the short term, there is a larger effect for musculoskeletal manual approaches manipulations compared to other conservative treatments for TMD.
McNeely et al., 2006 [48]	12 RCTs (*n* = 480)	To evaluate the methodological quality of, and summarize the evidence from, randomized controlled trials (RCTs) that examined the effectiveness of PT interventions in the management of TMD.	Mean Age (y): NA (18–76) Diagnosis: Muscular, articular, or mixed TMD according to RDC/TMD or authors own diagnosis criteria.	Intervention groups: TE alone, MT alone, TE + MT, acupuncture and electrophysical modalities Comparison groups: Other treatment and sham.	No MA	PI: Pain scale (0–5), 6-poit pain scale (0–6), VAS (0–10) and NRS (0–10) Pain-free MMO (mm): Ruler LE (mm): Ruler PPT (kg/cm^2^/s): Algometer Clinical disfunction score	Posture training in combination with other therapies on myogenous TMD reported significant improvements in PI and MMO, PPT, and the modified SSI, in favor of the addition of postural exercise training. Significant improvement in PI and MMO in favor of the MT + TE vs. occlusal ST on anteriorly displaced temporomandibular disks in patients with articular TMD. PRFE therapy was not found to be significantly better than sham PRFE for articular TMD. TENS were not significantly better than muscular awareness relaxation therapy, sham TENS, or BF. Significant improvements were found in MMO and EMG activity for the muscular awareness relaxation therapy group when compared with treatment with TENS and sham TENS. BF was not found to be significantly better in reducing PI when compared with relaxation therapy or occlusal ST. However, it did result in significant improvement in MMO when compared to occlusal ST. LLLT showed significant improvements in active and passive MMO and in LE, compared with sham laser. However, no significant differences were found in PI reduction between the groups. Authors’ conclusions: The results of this systematic review support the use of active and passive oral exercises and exercises to improve posture as effective interventions to reduce symptoms associated with TMD. There is inadequate information to either support or refute the use of acupuncture in the treatment of TMD. There is no evidence to support the use of electrophysical modalities to reduce TMD pain; however, the evidence suggests improvements in MMO may result from treatment with muscular awareness relaxation therapy, BF training, and LLLT treatment. Most of the studies included in this review were of very poor methodological quality; therefore, these findings must be interpreted with caution.
Medlicott et al., 2006 [49]	22 RCTs (*n* = 924) 8 not-RCTs	Analyze the effectiveness of PT interventions for TMD.	Mean Age (y): NA Diagnosis: Muscular TMD, DDWR, Arthralgia, arthritis, and arthrosis according to RDC/TMD.	Intervention groups: TE, MT, electrotherapy, relaxation training, BF and TE + electrotherapy Comparison groups: No treatment, placebo and ST.	No MA	PI: VAS PPT (kg/cm^2^/s): Algometer MFIQ Modified SSI MMO (mm): NA LE (mm): NA	TE and MT, alone or in combination, may be effective in the short term in increasing MMO in people with TMD resulting from acute disk displacement, acute arthritis, or acute or chronic myofascial TMD. Mid-laser therapy may decrease PI and improve MMO and LE in people with TMD secondary to acute disk displacement and may be more effective than other electrotherapy modalities in the short term. Relaxation techniques and BF, EMG training, proprioceptive re-education may be more effective than placebo treatment or occlusal ST in decreasing PI and increasing MMO in people with acute or chronic muscular TMD in the short term and the long term. Authors’ conclusions: Programs involving combinations of TE, MT, postural correction, and relaxation techniques may decrease PI and increase MMO in the short term in people with TMD resulting from acute disk displacement, acute arthritis, or acute myofascial TMD.
Melis et al., 2019 [22]	4 RCTs (*n* = 215)	To evaluate the efficacy of OMT for the treatment of TMD	Mean Age (y): NA Diagnosis: TMD according to NA.	Intervention groups: OMT + self-care, OMT, LLLT + OMT and LLLT + OME Comparison groups: Self-care, no treatment, stabilization appliance and sham LLLT.	No MA	PI: NRS, VRS, MPQ and SSI. MMO (mm): NA Head posture: Horizontal distance of the tragus of the ear to the acromion, neck inclination and cranium rotation	This SR found a statistically and clinically significant decrease in PI, with no difference between the groups when comparing OMT + self-care vs. self-care alone, and a clinically non-significant change in head posture. When comparing OMT vs. no treatment, no difference in PI was seen, but there was a significant difference in otologic and orofacial symptoms. When comparing OMT vs. stabilization appliance, PI in both groups decreased, but there were better results in the OMT treatment group. When comparing LLLT + OME, OMT, placebo LLLT + OME and LLLT, general decrease in PI in all groups, with stability at 3-month follow-up. LLLT + OMT/OME was more effective than LLLT alone, both for reducing TMD symptoms and to rehabilitate orofacial function. Authors’ conclusions: Even though scientific evidence is weak because of the limited number and low quality of RCTs available in the literature, OMT, similar to other conservative and reversible treatments, has favorable cost–benefit and risk–benefit ratios; therefore, such therapy can be advised for patients with TMD and associated orofacial myofunctional disorders.
Munguia et al., 2018 [62]	8 RCTs (*n* = 255)	To determine the efficacy of LLLT in treating myofascial TMD compared to placebo.	Mean Age (y): NA (16–60) Diagnosis: Myofascial TMD according to RDC/TMD and six myofascial pain syndrome criteria.	Intervention groups: LLLT Comparison groups: Sham LLLT.	Change and differences in PI at the end of treatment: 6 (*n* = 176) Change and differences in PI at 3–4 weeks follow-up: 5 (*n* = 160) MMO just after treatment: 2 (*n* = 59) MMO at 1 month follow-up: 2 (*n* = 59)	PI: VAS MMO (mm): NA	MA showed that LLLT vs. placebo provided a significantly greater reduction for PI from baseline to the end of the treatment with a moderate quality of evidence, and for PI after 3 to 4 weeks follow-up (SDM = –1.405 [−2.611 to −0.199]; *p* = 0.022; I^2^ = 91%), with a moderate quality of evidence. Improvement in the LLLT group after treatment was on average 2.2 points better on a VAS (0–10), so the improvements in this review may be clinically significant. LLLT provided a non-significant increase in MMO just after treatment compared to placebo, with a low quality of evidence, and a significant increase in MMO at 1 month after treatment (SDM = 0.686 [0.151, 1.220]; *p* = 0.012; I^2^ = 0%), with a low quality of evidence. Authors’ conclusions: LLLT seems to be effective in reducing PI in patients with myofascial TMD with moderate-quality evidence and increasing MMO with low-quality evidence. However, due to the high heterogeneity, small number of studies, and high risk of bias of the included studies, the results are not definitive, and further well-designed studies are needed.
Paço et al., 2016 [63]	7 RTCs (*n* = 329)	To analyze the effects of PT management of TMD.	Mean Age (y): NA Diagnosis: TMD according to RDC/TMD, patients’ medical history and Rx and medical and dental examination.	Intervention groups: MT + exercise, MT + house PT, MT alone and DN Comparison groups: ST, control, education, self-care, sham.	Any PT treatment vs. other intervention/placebo/no intervention on PI at rest: 6 (*n* = 317) Any PT treatment vs. other intervention/placebo/no intervention on MMO: 7 (*n* = 329)	PI: VAS, MPQ, 11-point GCPS and NPS Mandibular Function: MFIQ Active MMO (mm): NA Passive MMO (mm): NA PPT (kg/cm^2^/s): Algometer	The 1/3 studies assessed found a significant PI reduction at rest while the other 2/3 found no significant differences between the PT and control groups. Two studies found a significant difference between the intraoral myofascial therapy (IMT) and education and self-care groups that favored the IMT group, although it was not clinically significant. One study showed significant differences favoring DN vs. sham group. Two studies assessed no significant differences in MPQ between the PT and control groups. The 1/3 studies (using DN) assessed improvements over the masseters muscle PPT. Two studies assessed passive MMO and found no differences between groups. On active MMO, one study showed a significant increase in the experimental group and three studies found no differences between the physiotherapy and control groups. The MA showed that PT treatment vs. other intervention/placebo/no intervention on PI at rest was a statistically significant better favoring intervention [SMD = −0.63 (−0.95 to −0.31); 95% CI, −0.95 to −0.31; heterogeneity: I^2^ = 0.0%. *p* = 0.447; Egger’s test: *p* = 0.264]. When the analysis was performed restricted to studies that presented the same diagnostic criteria, the estimated summary remained similar. There was an improvement in MMO favoring intervention, although the differences found were not significant [SMD = 0.33 (−0.07 to 0.72); 95% CI; I^2^ = 61.9%; *p* = 0.007; Egger’s test: *p* = 0.575]. When the analysis was performed restricted to studies that presented the same diagnostic criteria, the estimated summary remained similar. Authors’ conclusions: In this review, PT interventions were more effective than other treatment modalities and sham treatment in the management of TMD for PI reduction, and there was a tendency toward improved for active MMO. However, these results are not definitive and should be interpreted with caution, mostly due to the small number of included studies and to the variability of the instruments used to assess the outcomes.
Pretucci et al., 2011 [64]	6 RTCs (*n* = 191)	To assess the scientific evidence on the efficacy of LLLT in the treatment of TMD.	Mean Age (y): NA (20–68) Diagnosis: TMD according to Anamnesis, muscle palpation, TMJ palpation, TMJ auscultation, radiographs, and MRI.	Intervention group: LLLT Control group: Sham LLLT.	LLLT vs. placebo on PI: 6 (*n* = 191) LLLT vs. placebo on MMO and LE: 2 (*n* = 65)	PI: VAS (mm) MMO (mm): NA Left LE (mm): NA Right LE (mm): NA	Two RCTs reported a non-significant difference in PI reduction between pre- and post-treatment, while the difference was statistically significant in the remaining four trials. The WMD for the MMO differences in baseline–end between LLLT and placebo groups was 4.04 mm and those for right LE and left LE were 1.64 and 1.90 mm, respectively. The MA showed that there was a reduction in PI for LLLT, but the MD of PI reduction was not statistically significant in comparison to placebo [MD: 7.77 (−2.49 to 18.02); 95% CI; heterogeneity: tau^2^ = 85.74; x^2^ = 11.08, df = 5 (*p* = 0.05); I^2^ = 55%; Test for overall effect: Z = 1.48 (*p* = 0.14)]. There was a significant difference in MMO with no heterogeneity in the LLLT group vs. placebo [MD: 4.04 (3.06 to 5.02); 95% CI; heterogeneity: Tau^2^ = 0.00; x^2^ = 0.04, df = 1 (*p* = 0.84); I^2^ = 0%; test for overall effect: Z = 8.10 (*p* = 0.00001)]. There was a significant difference in right LE with high heterogeneity in the LLLT group vs. placebo and there was a non-significant difference with high heterogeneity in left LE in the LLLT group vs. placebo. Authors’ conclusions: The results of this systematic review and MA indicated that there is no evidence to support the use of LLLT in the treatment of TMD.
Randhawa et al., 2016 [50]	7 RCTs (*n* = 753)	To investigate the effectiveness of non-invasive interventions compared with other intervention, placebo/sham interventions, or no intervention in improving self-rated recovery, functional recovery, or clinical outcomes in adults and children with TMD.	Mean Age (y): 44 (18–70) Diagnosis: Persistent TMD (>3 months) or TMD of variable duration according to the RDC/TMD axis II, GCPS score (0, I, II low, II high, III or IV), self-report pain and tenderness of the muscles of mastication and TMJ region and limited mandibular movements.	Intervention groups: Non-invasive interventions Comparison groups: Other interventions, placebo/sham interventions or no interventions	No MA	PI: VAS and GCPS MMO (mm): NA	There were statistically significant between-group differences at 12 months for PI favoring structured self-care management vs. usual treatment. At the 6-month follow-up, participants in the IMT group reported greater improvement in jaw PI at rest, jaw PI upon MMO, and jaw PI upon clenching. However, there were no differences in PI between IMT with structured education and TMD stretching compared with IMT alone. Authors’ conclusions: Our review suggests that patients with persistent TMD may benefit from self-care management, or IMT. The current evidence does not support the use of occlusal device to reduce pain and disability in patients with TMD. Other conservative interventions for the management of TMD have not been supported by studies with a low risk of bias.
Melo et al., 2018 [51]	1 RCT (*n* = 70 women) 2 Not-RCT (*n* = 64 women)	To evaluate systematically the evidence of the efficacy of Global Postural re-education in the treatment of pain in individuals with TMD.	Mean Age (y): 22.56 ± 3.40 to 36.2 ± 9.8 Diagnosis: TMD according to Helkimo index or DC/TMD.	Intervention groups: Global postural re-education (type of TE) Comparison groups: No treatment or static segmental stretching.	No MA	PI: VAS or DC/TMD (Axis 1) PPT (kg/cm^2^/s): Algometer	All included studies found the same positive effects in the RPG and the static segmental stretching groups compared to the control groups in the short term. The effects seem to diminish in the medium term. Authors’ conclusions: It was possible to conclude that GPRS to be effective in reducing pain present in TMD, in which it was emphasized the treatment of muscle chains added to body awareness and breathing. Considering the results obtained in this review, it is evident that there is no superiority between GPR, postural exercises and static segmental stretching. However, more randomized clinical trials with greater methodological rigor are needed.
Tunér et al., 2019 [51]	39 RCTs (1391)	To review all documents regarding PBMT application in TMD patients and to suggest a preliminary evidence-based protocol for PBMT administration for these patients.	Mean Age (y): NA Diagnosis: TMD according to NA.	Intervention groups: PBMT Comparison groups: Placebo.	No MA	PI: VAS Mandibular function: CMI MMO (mm): NA PPT: NA EMG: NA	The findings of the included articles suggested that PBMT was an effective way of decreasing PI as compared with placebo in TMD patients (29 studies out of 39). Moreover, PBM improved mandibular movements in eight studies and reduced patients’ anxiety in two studies. Collecting information of these heterogeneous RTCs showed beneficial impacts of PBM on reducing PI at the short-term follow-up (10 days to 4 weeks). Functionality of TMJ was assessed in terms of MMO, EMG and CMI, indicating that the total effect favored PBM in comparison with placebo. Moreover, four studies indicated PPT improved by PBM. Authors’ conclusions: Despite the observed limitation of PMBT, it seems that PBM can relieve PI in TMD patients and improve mandibular functionality. Given the high discrepancy within the included studies in this review, we highlight the need for further precise RCTs with larger sample sizes to assess its efficacy.
Lanas-Teran et al., 2019 [53]	13 RCTs (*n* = 546)	To establish whether there is evidence that LLLT can reduce the main symptoms of TMD and to determine the most effective application protocol.	Mean Age (y): NA Diagnosis: Mixed TMD according to NA.	Intervention groups: LLLT Comparison groups: Other laser types, other treatments, or ST	No MA	PI: NA MMO: NA Masticatory difficulty: adapted VAS Jaw movements (mm): PE and LE	An energy density of 3 J/cm^2^ decreased PI in the groups treated by LLLT but most authors used energy densities of 4 J/cm² and 8 J/cm², also with favorable results in terms of improvement in TMD symptoms. In some studies, groups treated with lasers showed an improvement in pain of up to 4 fold the pain levels of the placebo group. However, some authors found no significant difference between scores in this outcome after 4 weeks. Some authors evaluated jaw movements in terms of PE and LE, finding out an increase in dimensions of these jaw movements after LLLT. Authors’ conclusions: LLLT may be considered an alternative in the relief of symptoms of clinical TMD manifestations; however, there is no evidence of one protocol being superior to all the others.
Van der Meer et al., 2020 [65] (Corrigendum data; PMID: 33622659)	5 RCTs (*n* = 220)	To evaluate the effectiveness of PT on concomitant headache PI in patients with TMD.	Mean Age (y): NA (28–36) Diagnosis: Headache + TMD according to DC/TMD.	Intervention groups: PT Comparison groups: Counseling, ST, global postural re-education, education, and MT.	Overall PT vs. control on headache PI: 5 (*n* = 220) Counseling + exercise vs. counselling and/or ST on headache PI: 3 (*n* = 153)	Headache PI: VAS and colored analog scale	When comparing static stretching vs. global stretching (Global postural re-education) in one study, there was a large effect size in favor of static stretching on reducing headache PI with a low quality of evidence. When comparing orofacial and cervical MT vs. cervical MT in one study, orofacial and cervical MT was superior to cervical MT alone, reducing headache PI, with a low quality of evidence. MA showed that when comparing PT vs. control on headache PI, PT had a small overall effect, but not significant, on reducing headache PI, with a very low quality of evidence. When comparing counselling + TE vs. counselling and/or ST on headache PI, there were no difference in effects, with a low quality of evidence. Authors’ conclusions: PT interventions presented a small, but not significant, effect on reducing headache PI in subjects with TMD, with a low level of certainty. As orofacial PT and cervical MT do appear to be effective to reduce headache PI, a specialized physical therapist should be part of the health care team for the treatment of TMD and headache, although they may not be available in all countries.
Vier et al., 2019 [66]	7 RCTs (*n* = 199)	To investigate the effects of DN on orofacial pain of myofascial pain origin in patients with TMD.	Mean Age (y): NA (>18) Diagnosis: Myofascial TMD according to RDC/TMD, Helkimo and HIS.	Intervention groups: DN Comparison groups: Placebo, sham, and other interventions.	DN vs. Sham in PI: 2 (*n* = 70) DN vs. Sham in PPT: 3 (*n* = 82) DN vs. Sham in Pain free MMO: 2 (*n* = 62) DN vs. other interventions in PI: 2 (*n* = 68) DN vs. other interventions in PPT: 2 (*n* = 36)	PI: VAS and NRS PPT (kg/cm^2^/s): Algometer Pain-free MMO (mm): Millimeter ruler and caliper	MA showed not statistically significant difference between DN vs. sham on PI [MD = 0.30 (−0.83, 1.43); Tau2 = 0.24; Chi 2 = 1.32; df = 1 (*p* = 0.25); I2 =24%; Z = 0.52 (*p* = 0.60)], better results for DN vs. other interventions on PI [MD = −0.74 (−1.25, −0.22); Chi2 = 1.32; df = 1 (*p* = 0.25); I2 = 24%; Z = 2.80 (*p* = 0.005)], better results for DN vs. sham for PPT [MD = −0.56 (−0.81, −0.31); Tau2 = 0.03; Chi2 = 4.41; df = 2 (*p* = 0.11); I2 = 55%; Z = 4.40 (*p* < 0.0001)], not statistically significant difference between DN vs. other interventions for PPT [MD = 0.08 (−0.12, 0.27); Chi2 = 0.17; df = 1 (*p* = 0.68); I2 = 0%; Z = 0.79 (*p* = 0.43)] and not statistically significant difference between DN vs. sham for pain-free MMO [MD = −0.12 (−3.04, 2.80); Tau2 = 1.78; Chi2 = 1.66; df = 1 (*p* = 0.20); I2 = 40%; Z = 0.08 (*p* = 0.93)] in the short term, with a very low quality of evidence. Authors’ conclusions: DN is better than other interventions for PI as well as than sham therapy on PPT, but there is very low-quality evidence and a small effect size.
Xu et al., 2018 [67]	31 RCTs (*n* = 963)	To evaluate the effect of LLLT versus placebo in patients with TMD.	Mean Age (y): NA Diagnosis: TMD according to NA.	Intervention groups: LLLT Comparison groups: Sham LLLT.	MA of LLLT vs. Placebo: PI at the final follow-up time point: 17 (*n* = 643) Mean difference (MD)of PI between the baseline and the final follow-up time point: 19 (*n* = 679) Active MMO at the final follow-up point: 9 (*n* = 301) Passive MMO at the final follow-up time point: 3 (*n* = 144) LE at the final follow-up time point: 12 (*n* = 477) PE at the final follow-up time point: 5 (*n* = 157)	PI: VAS Active MMO (mm): NA Passive MMO (mm): NA LE (mm): NA PE (mm): NA PPT (kg/cm^2^ or kpa or mm): Algometer	MA showed statistically significant reduction in PI at the final follow-up time point favored LLLT vs. placebo when combining any LLLT dose and any follow-up time point. Subgroup analysis showed significant differences between LLLT and placebo groups at high dosage and unknown dosage. However, there were no significant differences between the two groups at low dosage. There were significant differences between the two groups at the short-term follow-up (<2 weeks). However, LLLT failed to show significant favorable effects on PI scores at long-term follow up (>2 weeks) compared to placebo (WMD = −14.84; 95% CI = −35.35–5.68; *p =* 0.16; I^2^ =97%). The overall effect favored LLLT over placebo in active MMO (WMD = 6.37 [2.82, 9.93]; *p* = 0.0004; *I*2 = 95%), LE, and PE. For PPT, it was impossible to estimate the overall effect size across the different scales. Four studies showed a significant change, while two reported no change of PPT in the LLLT group compared to the placebo group. Authors’ conclusions: The overall effect illustrated that LLLT effectively relieves PI over placebo and improves functional outcomes such as MMO in TMD patients, in the short term (< 2 weeks).
Zwiri et al., 2020 [54]	32 studies (*n* = 1172) 25 RCTs (*n* = NA) and 7 Non-RCTs (*n* = NA)	To evaluate the effectiveness of LLLT application in TMD and review the evidence from previous studies with their sample size and methodology in the management of TMD.	Mean Age (y): NA (15–73) Diagnosis: TMD according to NA.	Intervention groups: LLLT Comparison groups: Conventional treatment modalities.	No MA	PI: NA MMO (mm): NA	In this systematic review, 25/32 studies reported a reduction in PI compared with conventional treatment, while 7/32 studies did not find any significant difference, being the Ga-Al-As (LLLT) laser with a variation of 780–904 nm wavelength the most used to treat the TMD patients in the studies. Authors’ conclusions: After this review, LLLT can be recommended as a beneficial treatment approach for TMD patients.

Abbreviations: Al: Aluminum; As: Arsenium; BF: Biofeedback; BTX: Botulinum toxin; CBT: Cognitive behavioral therapy; CMI: Craniomandibular Index; DAL: Daily activity limitation; DC/TMD: Diagnostic Criteria for Temporomandibular Disorders; DDWR: Disc displacement with reduction; DN: Dry needling; EMG: Electromyography; Ga: Gallium; GCPS: Graded chronic pain scale; AIA: Intra-articular injection; IMT: Intraoral myofascial therapy; LE: Lateral excursion; LLLT: Low-level laser therapy; MA: Meta-analysis; MD: Mean difference; MFIQ: Mandibular function impairment questionnaire; MMO: Maximum mouth opening; MPQ: McGill pain questionnaire; MRI: Magnetic resonance imaging; MT: Manual therapy; NA: Not available; NRS: Numeric rating scale; NSAID: Non-steroidal anti-inflammatory drug; OME: Oral myofunctional exercise; OMT: Oral myofunctional therapy; PBMT: Photobiomodulation therapy; PE: Protrusion excursion; PI: Pain intensity; PPT: Pain pressure threshold; PRFE: Pulsed radio-frequency energy; PRP: Platelet-rich plasma; PT: Physical therapy; RCT: Randomized controlled trial; RDC/TMD: Research Diagnostic Criteria for Temporomandibular Disorders; ROM: Range of motion; SSI: Symptom severity index; ST: Splint therapy; TE: Therapeutic exercise; TENS: Transcutaneous electrical nerve stimulation; TMD: Temporomandibular disorders; TMJ: Temporomandibular joint; TTH: Tension-type headache; US: Ultrasound therapy; VAS: Visual analogue scale; VRS: Verbal rating scale; WMD: Weighted mean difference; Y: years.

**Table 2:** Intervention’s characteristics of the included systematic reviews.

**Study**	**Group**	**Type of Intervention**	Intervention Description	Characteristics of the Session (Treatment Time, Dosage, Intensity)	Total Intervention Time, Frequency of Sessions per Week and/or Day, and Total Number of Sessions	Follow-Up Time Points
Al-Moraissi et al., 2020 [55]	Intervention group:	DN, acupuncture, WN	DN: It refers to direct needling consisting of dry needling, with thin monofilament needle without any chemical agent injected directly (superficial or deep) into the masticatory muscles. Acupuncture: Penetration of the dry needling, thin filiform needles into proximal- and distal-specific acupoints according to Western biomedical acupuncture and traditional Chinese medicine. WN: Intramuscular injections in the masticatory muscles. Substances used were BTX, lidocaine, granisetron, mepivacaine, collagen and platelet-rich plasma.	Total session time: NA Intensity: NA Dosage: NA	Total intervention time: NA Frequency of sessions per week and/or day: NA Total number of sessions: From 1 to 4	Immediate post-treatment, 3 weeks, 1 month, and 6 months
	Comparison group:	Placebo				
Al-Moraissi et al., 2020 [56]	Intervention group:	Conservative treatment, intra-articular injections of HA or CS, arthroscopy alone, arthrocentesis with or without HA, CS and PRP, arthroscopy with or without HA and PRP, and open joint surgery	Conservative treatment: Muscle exercises and occlusal ST. Arthroscopy alone: Included lysis and lavage using normal saline or Ringer solution without injection of any medication. Open joint surgery: Which includes discectomy, high condylectomy, disc repositioning and arthroplasty.	Total session time: NA Intensity: NA Dosage: NA	Total intervention time: NA Frequency of sessions per week and/or day: NA Total number of sessions: NA	5 months, 6 months, and 4 years
	Comparison group:	Intra-articular injection (IAI) of normal saline, inactive laser				
Alves et al., 2013 [53]	Intervention group:	MT + physical medicine	MT (Mandibular Manipulation): Forcing the mandible with consecutive movements, inferior, anterior, superior, and posterior. Physical medicine: Which included mandibular manipulation, occlusal techniques, and NSAIDs.	Total session time: NA Intensity: NA Dosage: NA	Total intervention time: NA Frequency of sessions per week and/or day: NA Total number of sessions: NA	1 week, 1 month, 2 months, 3 months, 6 months, 12 months, 18 months, 24 months, and 60 months
	Comparison group:	Medical management, arthroscopic surgery, arthroplasty, palliative care, and controls				
Armijo-Olivo et., al 2016 [15]	Intervention group:	TE, MT, TE + other interventions, and MT + TE	TE: Any kind of exercises as: posture correction exercises, general jaw exercises alone or in combined with a neck exercise program, jaw/neck exercise alone or as part of a conservative intervention. MT: Facial manipulation, intra-oral myofascial therapy, mobilization of the cervical spine mobilization of atlantoaxial joint, manipulation of the upper thoracic spine, massage to masticatory muscles, mobilizations of TMJ joint. TE + other interventions: Jaw/neck exercises alone or combined with other therapies such as medications, surgery or selfcare recommendations. MT + TE: MT + jaw/neck exercise program, MT + stretching techniques.	Total session time: 12 min–1 h Intensity: NA Dosage: NA	Total intervention time: From 2 weeks to 12 months Frequency of sessions per week and/or day: 3–6 times/day 1–3 sessions/week Total number of sessions: NA	Immediately post-treatment, 4 weeks, 6 weeks, 2 months, 3 months, 6 months, 9 months, 12 months
	Comparison group:	ST, medication, auriculotemporal nerve block, arthroscopy, arthroplasty, medical self-care, placebo, waiting list control, standard care, BTX				
Brochado et al., 2019 [16]	Intervention group:	Occlusal ST, LLLT, PBMT, US, TENS, MT, TE (oral exercises), TEd (behavioral education), and acupuncture	LLLT: Application of light within the red and near infra-red wavelength range of 600–1000 nm. US: Application of mechanical vibrations at frequencies above 16 Hz generated by a piezoelectric effect using a frequency between 1.0 and 3.0 MHz. TENS: Modulates the control system of endogenous pain. MT: NA. Behavioral education: Any type of self-management or self-care that includes cognitive behavioral techniques as education about negative habits and counseling, relaxation techniques, and home exercises. Oral exercises: Any type of stretching and strengthening masticatory muscles and relaxation exercises, increasing mobility, tissue regeneration and helping patients to overcome the fear of moving the TMJ.	Total session time: NA Intensity: NA Dosage: NA	Total intervention time: NA Frequency of sessions per week and/or day: NA Total number of sessions: NA	Immediately post-treatment
	Comparison group:	Other treatments, placebo/sham, no treatment				
Calixtre et al., 2015 [17]	Intervention group:	MT	MT: Intra-oral myofascial release techniques, massage therapy on masticatory muscles, atlanto-occipital joint thrust manipulation, thoracic manipulation, and mobilization for the upper cervical spine.	Total session time: NA Intensity: NA Dosage: mobilization velocity (0.5 Hz).	Total intervention time: NA Frequency of sessions per week and/or day: NA Total number of sessions: From 1 to 10 sessions	Immediately post-treatment, 2 days, 3 days, 4 weeks, 5 weeks, 6 weeks, 2 months, 6 months, 12 months
	Comparison group:	Placebo, standard treatment, other treatments				
Chen et al., 2015 [18]	Intervention group:	LLLT	LLLT: Is a non-thermal type of light, thought to reduce inflammation through inhibition of PEG2 formation and suppression of cyclooxygenase 2. Types of LLLT used: GaAIAs, Nd: YAG, HeNe with wavelength from 632 to 1064 nm.	Total session time: 10–180 s Intensity: Wavelength: 632–1064 nm Energy per point: 0.3–480 (J/point per session) Dosage: Energy density: 1.5–112.5 J × cm^2^	Total intervention time: NA Frequency of sessions per week and/or day: From 1 to 12 sessions/week Total number of sessions: From 3 to 20 sessions	1 and 3 months
	Comparison group:	Placebo (sham therapy)				
de Melo et al., 2020 [46]	Intervention group:	MT + TE, and MT + TE + counseling	MT: Mobilization of the TMJ and/or soft tissues of the masticatory muscles and/or massage. TE: Passive or active stretching exercises, isometric tension against resistance exercises, and guided opening and closing of the mandibular movements.	Total session time: 10–50 min Intensity: NA Dosage: Stretching: 1 min, and coordination exercises: 20 repetitions	Total intervention time: 4 weeks Frequency of sessions per week and/or day: 3 sessions/week Total number of sessions: NA	4 weeks, 6 weeks, 3 months, 6 months, and 1 year
	Comparison group:	BTX injections, home physical therapy alone, no treatment, and counseling				
Dickerson et al., 2017 [19]	Intervention group:	MT + TE	MT: NA. TE: Intervention based on mobility, motor control, postural education, and mixed exercise therapy (combined interventions).	Total session time: 10–45 min Intensity: NA Dosage: Sets 5–10, repetitions 2–10, isometric exercises holding 10–30 s	Total intervention time: NA Frequency of sessions per week and/or day: 2–4 times/day, 1–2 sessions/week Total number of sessions: NA	10 min after treatment, 4 weeks, 17 weeks, and 52 weeks
	Comparison group:	Another type of treatment, and placebo				
Florjanski et al., 2019 [45]	Intervention group:	TE (BFB training): visual BFB, vibratory BFB, audio BFB, and contingent electrical stimulation	EMG Biofeedback: Providing biological information from muscles to patients in real-time that would otherwise be unknown. Surface electrodes are placed on the skin to measure frequency, intensity, and duration of muscle contraction. In these interventions, electrodes were placed over the master muscle, temporalis muscle or both. BFB: Patient modifies muscle activity with self-control, based on a constant feedback of a registered signal. Audio, visual, and vibratory signals were used. Contingent electrical stimulation: Is a method, in which the device emits a non-painful electrical pulse to the chosen muscle region when EMG activity exceeds the individually determined threshold.	Total session time: 20 min–8 h Intensity: NA Dosage: NA	Total intervention time: 2 nights–12 weeks Frequency of sessions per week and/or day: NA Total number of sessions: 2–42 sessions	5 nights, and 2 weeks
	Comparison group:	TENS treatment, occlusal ST, and control group				
Fricton et al., 2009 [58]	Intervention group:	TE	TE: Jaw stretching, neck stretching, posture training, standardized physiotherapy exercise, repetitive range of motion exercise, exercise in combination with other treatment.	Total session time: NA Total session time: NA Intensity: NA Dosage: NA	Total intervention time: NA Frequency of session per week and/or day: NA Frequency of sessions per week and/or day: NA Total number of sessions: NA	Immediately post-treatment, 2, 3, 6, 9, and 12 months
	Comparison group:	Non-intervention, placebo exercises, self-management, and other treatments				
Herrera Valencia et al., 2020 [20]	Intervention group:	MT + TE	MT: Caudal mobilization of the TMJ, manual treatment of the temporal and masseter muscles, cervical region treatment, TMJ neurodynamics, TMJ manipulation, TMJ traction mobilization, lateral and medial pterygoid muscle, and sphenopalatine ganglion treatments. TE: Cervical muscles isometric exercises and/or opening TMJ exercises. TEd: Health education and good habits communicated during the session by the clinical therapist.	Total session time: 10–50 min Intensity: NA Dosage: NA	Total intervention time: 2–18 weeks Frequency of session per week and/or day: 0.5–2 sessions per week Total number of sessions: 2–24 sessions	3, 4, and 12 months
	Comparison group:	BTX injection, TEd, no treatment, TE, and ST				
Jing et al., 2020 [59]	Intervention group:	LLLT	LLLT: Application of LLLT using different laser types: GaAs, AlGasAs, GaAlAs, HeNe, Nd: YAG, and InGaAlP.	Total session time: 10–180 s Intensity: Wavelength: 632.8–1064 nm Dosage: Energy density: 1–105 J × cm ^−2^ Exposure time per point: 10 s–10 min	Total intervention time: From 2 weeks to 8 weeks Frequency of session per week and/or day: NA Total number of sessions: 1–20 sessions	1 month
	Comparison group:	Placebo, and control group				
La Touche et al., 2020 [49]	Intervention group:	TE and MT, in combination or in isolation	TE: Self-mobilization exercises related to jaw opening and closing or side-to-side movement. In addition, some interventions combined mobilization exercises with isometric strength exercises or specific jaw muscle stretching exercises. MT: Mobilization of the TMJ using traction and translation movements and massage techniques on the masseter and temporal muscle.	Total session time: NA Intensity: NA Dosage: NA	Total intervention time: From 1 to 8 weeks Frequency of sessions per week and/or day: 3–5 times/day Total number of sessions: NA	1 week and 52 weeks
	Comparison group:	Surgery, no treatment after surgical treatment, ST, and NSAIDs				
La Touche et al., 2020 [60]	Intervention group:	MT + TE	MT: Mobilizations or high velocity manipulations of the cervical region, TMJ mobilizations, neuromuscular and nerve tissues techniques in the craniomandibular region. TE: Muscle conditioning for the cervical region and coordination exercises for masticatory muscles and therapeutic exercises for the craniomandibular region.	Total session time: NA Intensity: NA Dosage: NA	Total intervention time: NA Frequency of sessions per week and/or day: NA Total number of sessions: NA	48 h, 5 weeks, 6 weeks, 3 months, and 6 months
	Comparison group:	Placebo, sham mobilizations, no intervention, cervical MT alone				
Machado et al., 2018 [21]	Intervention group:	DN, WN, LLLT and infrared laser therapy	DN: DN in trigger point or close to it, by means of different length needles. Wet needling: Injection of substances in the trigger point. Substances used were procaine 1%, lidocaine 0.25% or 0.5%, lidocaine 0.25% + 0.2 mL corticoid, 0.2 mL BTX, 1 mL, lidocaine 0.5% 1 ml, 0.2 mL botulinum toxin, 1 mL granisetron, and 0.2 mL ketamine. Laser: Infrared laser and LLLT (GaA1As).	Total session time: NA Intensity: Infrared laser: 795 nm at 80 mW power LLLT: GaA1As 100 mW Dosage: Infrared laser: 4 or 8 J/cm^2^ LLLT: 80 J/cm^2^	Total intervention time: NA Frequency of sessions per week and/or day: NA Total number of sessions: NA	30 min after infiltration, 24 h, 1 week, 2 weeks, 1 month, 2 months, 3 months, and 6 months
	Comparison group:	DN as placebo, local anesthetic, false needling, methocarbamol and paracetamol combination drug therapy, saline solution 0.9%, 0.7%, and fascial manipulation				
Martins et al., 2016 [61]	Intervention group:	MT	MT (musculoskeletal manual approaches): Techniques as distraction mobilization technique, passive traction and translation movements, massage in jaw elevator muscle, osteopathic manipulative treatment, atlanto-occipital thrust, intraoral myofascial therapy, accessory movements, tender-trigger point and muscle stretching and tissue, cervical and TMJ mobilization and TMJ stabilization.	Total session time: NA Total session time: NA Intensity: NA Dosage: Number of MT techniques varied from 1 to 5	Total intervention time: 1 day to 24 weeks Frequency of sessions per week and/or day: 1 to 3 times/week Total number of sessions: NA	NA
	Comparison group:	Home exercise massage, usual care, sham treatment, and ST				
McNeely et al., 2006 [48]	Intervention group:	TE + other therapy, MT + TE, traditional TE, conventional therapy + oral exercise device, Ted (CBT) + posture correction, and electrophysical modalities: PRFE, LLLT, TENS1, and biofeedback	TE: Any type of jaw exercises o postural correction exercises, with or without any device, to improve strength and mobility in the region. MT: Techniques of manual mobilization, to reduce pain and restore mobility. Ted (CBT): Education on chronic pain, stress reduction, and relaxation training.	Total session time: TE: 1–5 min MT: NA EFM: 30 min Intensity: NA Dosage: NA	Total intervention time: TE: 4 weeks to 12 months Electrophysical Modalities: 1 week to 4 months Frequency of sessions per week and/or day: TE: 2 session per week to 1 session per month EFM: 1 per week to 3 per week Total number of sessions: TE: 8–16 sessions EFM: 3–20 sessions	TE: 0 to 12 months Electrophysical Modalities: 0 to 1 month
	Comparison group:	Occlusal ST, no treatment, CBT, TMD self -management instructions alone, sham, relaxation therapy				
Medlicott et al., 2006 [49]	Intervention group:	TE, MT, electrotherapy, relaxation training, and TEd	TE: Masticatory and neck musculature chilling, stretching exercises, home-exercise program MMO against resistance, active ROM exercises, coordination exercises and postural correction exercises. MT: Manual mobilization and massage techniques. Electrotherapy: PRFE, microcurrent electrical neuromuscular stimulation, LLLT, short-wave diathermy, TENS and ultrasound. Relaxation training and TE: Breathing and postural relaxation techniques, muscle relaxation techniques, EMG Biofeedback, relaxation tape and stress management education.	Total session time: TE and MT: NA. Electrotherapy: 40 sec–30 min Intensity: NA Dosage: TENS: 2–100 Hz Ultrasound: pulsed, 0.08–0.5 W/cm^2^ LLLT: 830–904 nm RPFE: 250 kHz Relaxation training and TE: NA	Total intervention time: TE and MT: 2 weeks to 6 months Electrotherapy: 2 weeks to 6 weeks Relaxation training and TE: 3 weeks to 8 weeks Frequency of sessions per week and/or day: TE and MT: 1/3 to 3 sessions per week Electrotherapy: 1 to 3 sessions/week Relaxation training and TE: 1 to 2 sessions/week Total number of sessions: TE and MT: 1 to 18 sessions Electrotherapy: 2 to 12 sessions Relaxation training and TE: 3 to 8 sessions	Immediately post-treatment, and 12 months
	Comparison group:	No treatment, occlusal ST, waiting list control, sham/placebo techniques				
Melis et al., 2019 [22]	Intervention group:	TE (OMT) + self-care, TE (OMT), LLLT + OMT, LLLT + OME	TE (OMT): It is based on oral exercises, which included tongue, neck, shoulders, and jaw exercises. The list exercises were not always described, but the aim was to favor an increase in blood circulation and pain relief, mandibular posture and mobility without deviations, coordination of the muscles of the stomatognathic system and equilibration of the stomatognathic functions compatibly with dental occlusion.	Total session time: NA Intensity: NA Dosage: NA	Total intervention time: NA Frequency of sessions per week and/or day: NA Total number of sessions: 9–13 sessions	Immediately post-treatment, 1 week, 4 weeks, 19 weeks, and 7 months
	Comparison group:	Self-care, no treatment, stabilization appliance, placebo LLLT				
Melo et al., 2018 [51]	Intervention group:	TE (global postural re-education training)	TE (global postural re-education training): This method is based on composition of muscle chains and advocates the global stretching of the muscles that compose them, based on the principle that dysfunctions can arise due to the retractions of muscle chains present throughout the body.	Total session time: 15–45 min Intensity: NA Dosage: NA	Total intervention time: 2–2.5 months Frequency of sessions per week and/or day: 1–2 per week Total number of sessions: 8–16 sessions	NA
	Comparison group:	Static segmental stretching exercises, and no treatment				
Munguia et al., 2018 [62]	Intervention groups:	LLLT	LLLT: It refers to Class IIIb lasers with less than 600 mW of power. It improves local microcirculation, resulting in increased oxygen supply to the hypoxic cells related to the trigger point area. Different laser types were used: diode laser, GaAIAS, GaAlAs diode laser, YAG laser, In-Ga-Al-P, and infrared laser.	Total session time: 19 s to 10 min Intensity: NA Dosage: Laser energy density (J/cm^2^): from 6.4 to 105; power density (mW): from 50 to 250; pulsed (HZ) or continuous mode; 1500 or continuous mode	Total intervention time: 4–5 weeks Frequency of sessions per week and/or day: 2–3 sessions/week Total number of sessions: 4–10 sessions	Immediately post-treatment
	Comparison groups:	Sham laser (inactive laser)				
Paço et al., 2016 [63]	Intervention groups:	MT + TE, MT + home physical exercise, TE, MT, and DN	NA	Total session time: NA Intensity: NA Dosage: NA	Total intervention time: 1 day–6 weeks Frequency of sessions per week and/or day: NA Total number of sessions: NA	Immediately post-treatment, 6 weeks, 20 weeks, 24 weeks, 46 weeks, and 1 year
	Comparison groups:	ST, control group, TEd and self-care, home physical TE				
Petrucci et al., 2011 [64]	Intervention groups:	LLLT	LLLT: Is a non-thermal type of light that is considered that this effect is a consequence of the reduction in levels of prostaglandin E2, which is among the most important proinflammatory mediators. Reduction in prostaglandin E2 could be observed within a range of dose between 0.4 and 19 J and within a range of power density between 5 and 21.2 mW/cm^2^. Different laser types were used: GaAlAs, HeNe, and GaAs.	Total session time: 10–180 s Intensity: Maximum pulse: 17–500 mW Dosage: Power density: 3500–38887 mW/cm^2^ Dose: 0.3–10 J	Total intervention time: NA Frequency of sessions per week and/or day: 1–3 sessions/week Total number of sessions: 3–20 sessions	1 month
	Comparison groups:	Sham LLLT (placebo)				
Randhawa et al., 2016 [50]	Intervention groups:	Self-care management, MT (myofascial therapy), structured TEd	Structured self-care management: Education, guided reading with structured feedback, relaxation and stress management training, self-monitoring of signs and symptoms, development of a “personal TMD self-care plan,” supervised practice and reinforcement of dentist-prescribed self-care treatments, and maintenance and relapse prevention. MT (intraoral myofascial therapy): Intraoral temporalis release; intraoral medial and lateral pterygoid (origin) technique; and intraoral sphenopalatine ganglion technique. TEd (structured education): Patients were instructed on chewing technique and relaxation stretching. They also attended short lectures on basic TMJ anatomy, biomechanics, disk displacement and dysfunction, and the role of psychoemotional factors in TMD.	Total session time: NA Intensity: NA Dosage: NA	Total intervention time: 5 weeks–2.5 months Frequency of sessions per week and/or day: 0.3–2 sessions/week Total number of sessions: 3–10 sessions	Post-intervention, 6 and 12 months
	Comparison groups:	Physiotherapy, education, medication, intraoral flat-plane occlusal appliances, and waiting list				
Turner et., 2019 [52]	Intervention groups:	LLLT	LLLT: Applied to TMJ and masticatory muscles.	Total session time: 10 s–20 min Intensity: NA Dosage: Energy density varied between 4 and 112.5 J/cm^2^	Total intervention time: NA Frequency of sessions per week and/or day: 1 sessions/week–5 sessions per week Total number sessions: 1–20 sessions	48 h, 10 and 12 days, 180 days, 3, 4, 5, 6 and 8 weeks and 12 months
	Comparison groups:	Placebo, and NSAIDs (piroxicam)				
Lanas-Teran et al., 2019 [53]	Intervention group:	LLLT	LLLT: Application of different laser types: GaAIAs 660–904 nm, Nd: Yang 1064 nm, HeNe 632.8 nm. LLLT: Due to their analgesic and anti-inflammatory effects, various types of lasers, such as HeNe and GaAlAs, are used in the management of TMD, each one being used at different wavelengths. The treatment is non-invasive, fast, and safe. Types of LLLT used: GaAIAs, Nd: Yang, HeNe.	Total session time: 10–180 s Intensity: Wavelength: GaAIas 660–904, Nd: Yang 1064, HeNe 632.8 nm Dosage: Energy density: 1.5–52.5 J × cm^−2^ Power density: 17–500 mW2	Total intervention time: NA Frequency of session per week and/or day: NA Total number of sessions: 3–20 sessions	Immediately post-treatment, and 180 days
	Comparison group:	Laser GaAIAs (780–830 nm), ST, sham laser), occlusal ST, and sham laser				
Van der Meer et al., 2020 [65]	Intervention groups:	Counseling + relaxation exercises + MT + TE + TEd	MT: Stretching + auto-massage jaw muscles, static stretching of cervical spine, upper limbs, and mandibular muscles. TE: Home exercises, jaw muscles and joint exercises. TEd: NA.	Total session time: From several minutes for home therapy to full 30 to 40 min sessions with a therapist Intensity: NA Dosage: NA	Total intervention time: NA Frequency of sessions per week and/or day: From daily for 3 months to weekly for 8 weeks Total number sessions: NA	From 2 weeks to 6 months
	Comparison groups:	Counseling + occlusal appliance, global posture re-education, TEd, ST, cervical MT				
Vier et al., 2019 [66]	Intervention groups:	DN	DN: Applied in masseter muscle, lateral pterygoid muscle, temporalis muscle, digastric muscle, splenius muscle, sternocleidomastoid muscle, and trapezius muscle. Other interventions: WN, LLLT, sham LLLT, and pain education.	Total session time: From 2 to 30 min Intensity: NA Dosage: NA	Total intervention time: 1–5 weeks Frequency of sessions per week and/or day: 1 session/week Total number of sessions: NA	From 24 h to 10 weeks
	Comparison groups:	Placebo/sham therapy, and other interventions				
Xu et al., 2018 [67]	Intervention group:	LLLT	LLLT: Application sites were generally the TMJ and/or temporomandibular muscles. Types of LLLT used: GaAlAs, GaAs, Nd: YAG, HeNe, InGaAlP, and diode laser.	Total session time: 10 s–45 min Intensity: Wavelength: 632.8–1064 nm Dosage: 1.5–112.5 J/cm^2^	Total intervention time: NA Frequency of sessions per week and/or day: 1–7 sessions/week Total number of sessions: 3–20 sessions	Immediately post-treatment, 3 months
	Comparison group:	Placebo, sham laser				
Zwiri et al., 2020 [54]	Intervention groups:	LLLT	LLLT: Has a low energy intensity and its effect is based on the light absorption process, which is between 630 and 1300 nm. The main impacts are bio stimulative, regenerative, analgesic and anti-inflammatory. Different types of LLLT used were Ga-Al-As, GaAs, semiconductor Ga-Al LLLT	Total session time: From 1.06 s to 8 min Dosage and intensity: Wavelength: 780 to 904 nm Energy: 1.5–144 J/cm^2^	Total intervention time: NA Frequency of sessions per week and/or day: 2–3 sessions per day for 1 week. 2–3 sessions per week for 4 weeks Total number of sessions of treatment: 6–24 sessions	NA
	Comparison groups:	NA				

Abbreviations: BFB: Biofeedback; BTX: Botulinum toxin; CBT: Cognitive behavioral therapy; CS: Corticosteroids; DN: Dry needling; GaAlAs: Arsenidegallium-aluminum; HA: Hyaluronic acid; HeNe: Helium-neon; LLLT: Low-level laser therapy; MT: Manual therapy; NA: Not available; NSAIDs: Non-steroidal anti-inflammatory drugs; OE: Oral exercises; OME: Oral myofunctional exercise; OMT: Oral myofunctional therapy; PBMT: Photobiomodulation therapy; PRP: Platelet rich plasma; PT: Physical therapy; ST: Splint therapy; TE: Therapeutic exercise; TEd: Therapeutic education; TENS: Transcutaneous electrical nerve stimulation; TMD: Temporomandibular disorders; TMJ: Temporomandibular joint; US: Ultrasound therapy; WD: Wet needling.

### 3.2. Methodological Quality Results

Following the recommendations of Shea et al. [31] for the use of the AMSTAR 2 instrument, individual item ratings were not combined to create an overall score; however, we analyzed the methodological quality based on critical and non-critical items.

The AMSTAR 2 scores of overall confidence in the results of the 31 SR ranged from “critically low” to “low”. Only five (16.1%) SR scored as “low” and were considered the highest-quality studies in this umbrella SR [15,55,60,64,65]. The critical items with the lowest scores were those related to the methods established prior to the performance of the review, the use of a comprehensive literature search strategy, providing a list of excluded primary studies, and justifying the exclusions. The critical items that were completed and marked as “yes” in more than 50% were items 9 (80.64%) and 13 (61.29%), in which the presence of a satisfactory technique for assessing the risk of bias in the primary studies is assessed, as well as the risk of bias itself when discussing the results of the review. The remaining critical items were not completed in more than half of the included studies, and none of the SR included reported the sources of funding for the primary studies included in each review.

Table 3 shows the results of the methodological quality assessment using the AMSTAR 2. The inter-rater reliability of the methodological quality assessment was high (k = 0.9).

### 3.3. Risk of Bias Results

**Figure 2:**
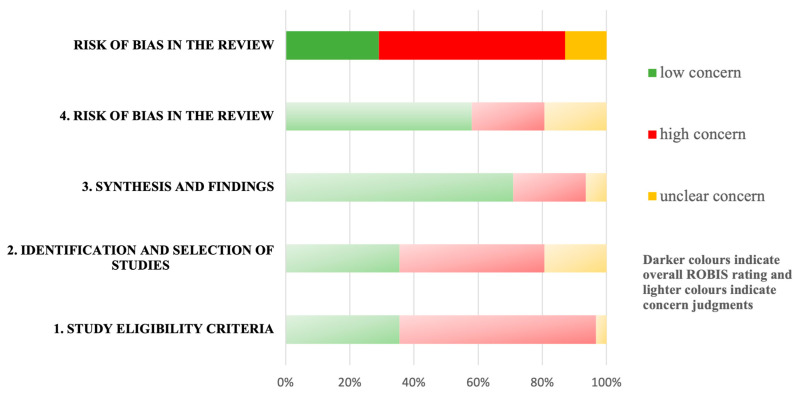
Risk of bias assessment with the ROBIS tool.

Table 4 and Figure 2 show the results of the risk of bias assessment using ROBIS. Of the 31 SR, 19 (61.3%) had a high risk of bias [16,18,19,20,45,46,50,51,52,53,54,58,59,61,62,63,64,65,66]; 10 (32.3%) had a low risk of bias [15,17,21,47,48,49,55,57,60,67]; and the remaining 2 had an unclear risk of bias [22,56].

The domains “Synthesis of Findings” and “Data Collection and Study Appraisal” had the lowest risk of bias, given that 24 (77.4%) SR scored with a “low concern” for both domains. In contrast, the domains “Study Eligibility Criteria” and “Identification and Selection of Studies” had the highest risk of bias, given that 21 (67.74%) and 19 (61.29%) of the SR, respectively, scored a “high concern” for both domains.

The inter-rater reliability for the risk of bias assessment was moderate (k = 0.6). We specifically analyzed the sources of funding for each SR, and only 9 of the 31 SR described that information [15,17,20,50,54,58,59,63,67]. Detailed information about the funding is shown in the Appendix, Table A3.

### 3.4. Evidence Map

**Figure 3:**
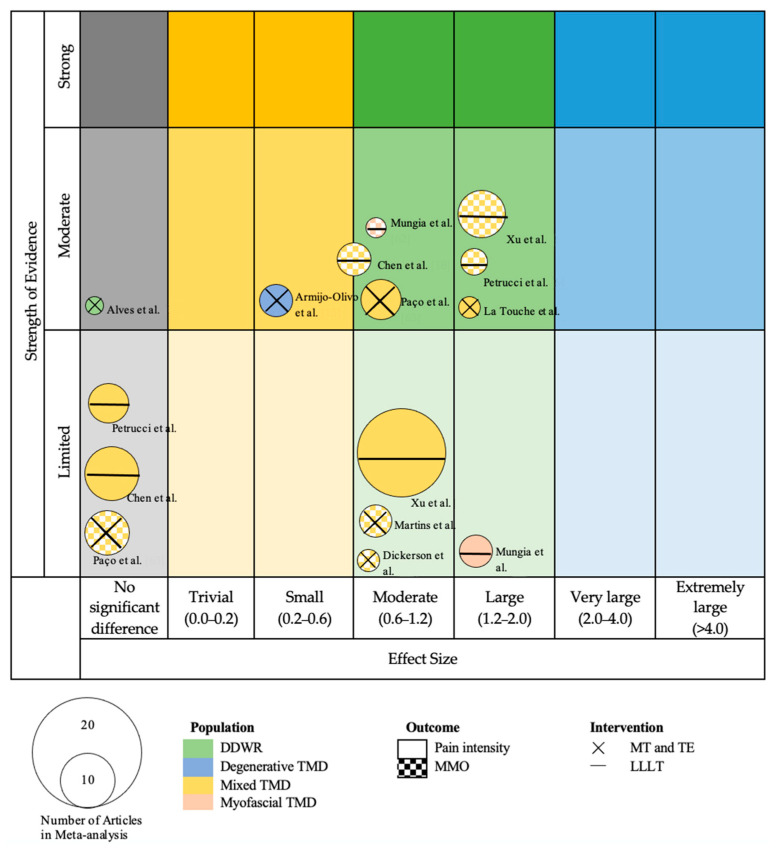
Mapping based on effect size of MMA and strength of the evidence. On the x-axis, the studies are classified according to the effect size of the meta-analysis with standard mean difference (SMD). On the y-axis, they are categorized according to the strength of evidence based on the Physical Activity Guidelines Advisory Committee (PAGAC) [15,18,19,57,60,61,62,63,64,67]. Green bubble: Disc displacement with reduction; blue bubble: degenerative temporomandibular disorder; yellow bubble: mixed temporomandibular disorder; orange bubble: myofascial temporomandibular disorder; X: manual therapy and therapeutic exercise; −: low-level laser therapy.

Figure 3 presents the results of the evidence map for the 10 SR included in the MMA.

### 3.5. Effects of Manual Therapy and Therapeutic Exercise Interventions

#### 3.5.1. Isolated Manual Therapy Interventions

Three SR examined isolated MT interventions and analyzed their effects on various types of TMD [17,57,61]. All of them found that MT alone, or in association with other conservative interventions, is an effective approach for reducing PI and increasing the MMO and pressure pain threshold in patients with TMD.

The types of MT studied were massage therapy, mobilization/manipulation of the TMJ, and mobilization/manipulation of the upper cervical spine, all of which showed positive effects on patients with TMD, except for the C7-T1 thrust manipulation intervention, which showed low evidence of non-significant differences when comparing the effects of placebo on the pressure pain threshold in 1 SR [17]. The MT intervention also showed the same beneficial effects as botulinum toxin (BTX) injection on MMO and PI in patients with TMD [17]. One SR studied MT interventions specifically focused on myofascial TMD and found that MT was superior to no treatment and confers greater benefits when added to home therapy than home therapy alone; however, it was not more efficient than BTX treatment [46]. When MT was combined with counselling techniques, its effectiveness was noticeably higher. However, MT alone was not superior to counselling techniques, and it did not confer greater benefits to patients with myofascial TMD when added to these techniques. Finally, only 1 SR analyzed the effects of MT on the treatment of patients with disc displacement without a reduction in the TMJ and found that, based on the analysis of 2 studies, at 8 weeks follow-up, the treatment of patients with manual TMJ therapy, nonsteroidal anti-inflammatory drugs (NSAIDs), and occlusal techniques was not superior to controls for PI and MMO. However, at 60 months follow-up, the conservative intervention had the same effects as the treatment with arthroscopic surgery and arthroplasty [57].

Isolated MT interventions consisted mainly of the application of intra- and extra-oral soft tissue techniques of the masticatory musculature and joint tissue techniques addressed to the TMJ and upper cervical spine, comparing their effect with standard medical treatment, including medication (NSAIDs) and/or oral splint or TMJ surgery, and sham MT or controls. Two SR analyzed the number of sessions applied in the treatment, which varied from 1 to 24 [17,61].

Finally, only 1 SR specifically analyzed the frequency of MT interventions and the number of MT techniques used, revealing that 1 to 5 MT techniques were used, and that the frequency of the sessions ranged from 1 to 3 sessions per week [61].

#### 3.5.2. Isolated Therapeutic Exercise Interventions

Three SR examined isolated TE interventions [22,51,58]. Two of the SR analyzed their effects on different combined types of TMD. One found that oral myofunctional therapy was not superior to controls or stabilization appliance (occlusal splint), it does not confer more benefits to self-care intervention, and it brings enhanced benefits to LLLT treatment [22]. In the other SR, positive effects were found for exercise therapy based on the emphasis on global exercises when added to body awareness and breathing techniques on reducing PI in the short term, with no superiority found between global postural re-education, postural exercises, and static segmental stretching [51]. Finally, only 1 SR analyzed the effects of TE interventions specifically on myofascial TMD and headache attributed to patients with TMD and found that TE based on stretching and postural relaxation was superior to controls and placebo on decreasing PI and headache frequency; however, the addition of a TE program to other conservative modalities did not show greater benefits in these outcome variables [58].

In terms of the interventions analyzed in the SR, isolated TE interventions consisted mainly of applying TMJ repetitive mobility exercises, cervical and TMJ motor control exercises, stretching exercises, and postural control exercise, comparing them with no treatment, self-management, and placebo exercises. Only 1 SR [22] included an analysis of TE interventions based on stomatognathic system functions and lingual exercises.

Two SR [22,51] analyzed the number of sessions applied in the treatment, which varied from 8 to 16. One SR [51] specifically analyzed the frequency of TE sessions and the duration of each session, finding that the frequency of the sessions ranged from 1 to 2 sessions per week, and the duration of each session varied from 15 to 45 min.

#### 3.5.3. Combined Manual Therapy and Therapeutic Exercise Interventions

Six SR examined MT and TE interventions in isolation or in combination [15,19,20,46,47,60]. All of them found positive results for MT and TE interventions on MMO and PI, and that the combined interventions could be beneficial and play a role in the TMD approach. Two SR found positive effects from the inclusion of MT and TE in the cervical region on PI and MMO. The first [15] found that MT and TE in the cervical spine had positive effects on PI and MMO in myofascial TMD; the second [60] found the same in myofascial TMD and mixed TMD; and also that the combination of a cervical intervention with a TMJ intervention had better results. Only 1 SR [47] analyzed the effect of MT and TE on disc displacement without reduction and found that the greatest benefits were found by combining both interventions.

In terms of the interventions analyzed in the SR, combined MT and TE interventions consisted mainly of applying soft tissue techniques on the masticatory muscles; articular mobilization, traction, and manipulation techniques on the TMJ and the cervical spine; TE based on motor control of the mandibular and cervical movements; repetitive opening exercises of the TMJ; isometric exercises of the masticatory and cervical muscles; and postural control exercises. Two SR [20,60] included interventions based on neuromuscular and nerve tissue techniques or TMJ neurodynamics, and only 1 SR [15] analyzed interventions including manipulation of the thoracic spine. Interventions were compared normally with splint therapy, medication, BTX, surgery, placebo or sham, and no treatment.

In the various SR, the total time of the sessions ranged from 10 to 60 min, the frequency of the intervention varied from 2 to 6 times per day and from 0.5 to 5 sessions per week, and the total intervention time was between 1 week and 12 months.

#### 3.5.4. Results of Quantitative Meta-Meta-Analysis of Combined Manual Therapy and Therapeutic Exercise Interventions

**Figure 4:**
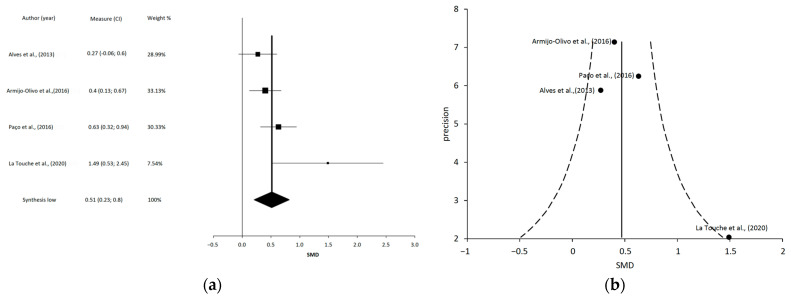

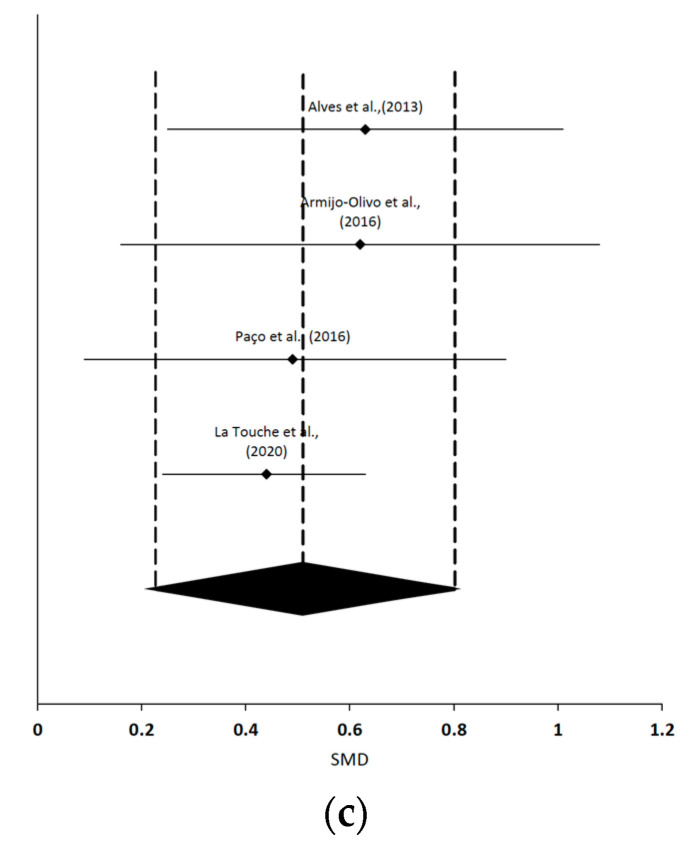
(**a**) The forest plot summarizes the results of included studies (standardized mean differences (SMD) and weight). The small squares represent the point estimate of the effect size and sample size. The lines on either side of the box represent a 95% confidence interval [15,57,60,63]. (**b**) Funnel plot aims to assess the existence of publication bias. (**c**) Sensitivity plot.

Regarding the quantitative analysis, the MMA of MT and TE on PI in patients with TMD revealed a statistically significant moderate effect size [15,57,60,63] (SMD = 0.51; 95% CI 0.8 to 0.23; *p* < 0.001). Given that the I^2^ was <75% (I^2^ = 57%), we considered that there was no evidence of heterogeneity. However, based on Cochran’s Q statistical test (Q = 6.97, *p* = 0.07), the heterogeneity among the reviews was considered significant. Although the shape of the funnel plot appeared to be asymmetrical in the dominant model, the sensitivity exclusion analysis suggested that no reviews significantly affected the pooled SMD. Forest, funnel and sensitivity plots are shown in Figure 4. The % of overlap in this MMA was 23.1% and the CAA = 0.07 (slight overlap). The overlapping of RCT is shown in Appendix, Table A4.

**Figure 5:**
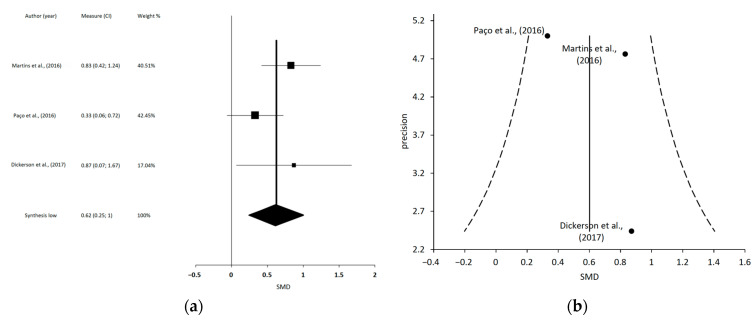

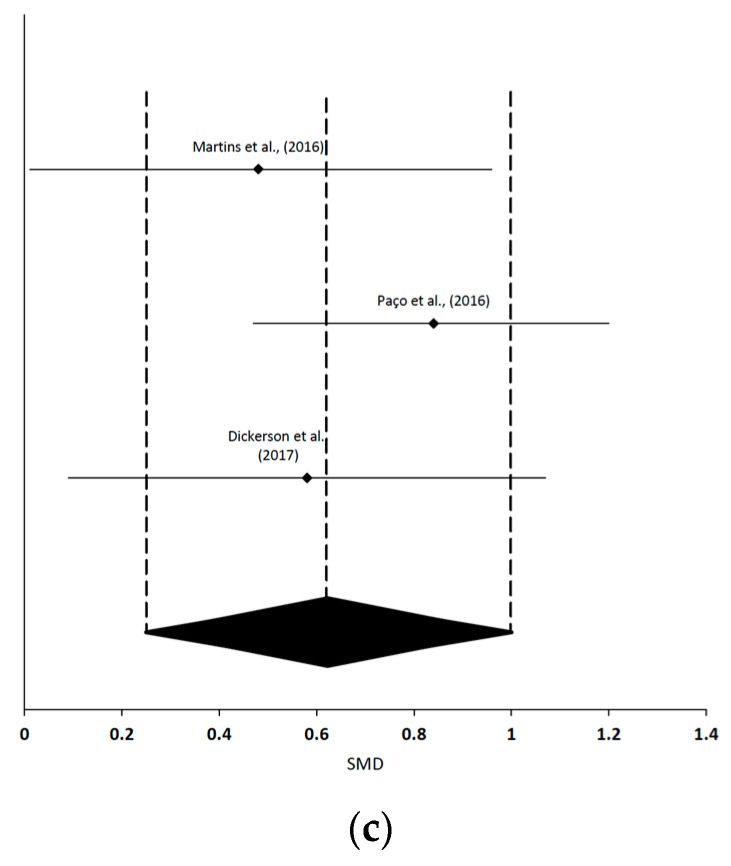
Synthesis forest, funnel and sensitivity plots of combined manual therapy and therapeutic exercise intervention on maximum mouth opening variable in patients with TMD [19,61,63]. (**a**) The forest plot summarizes the results of included studies (standardized mean differences (SMD) and weight). The small squares represent the point estimate of the effect size and sample size. The lines on either side of the box represent a 95% confidence interval. (**b**) Funnel plot aims to assess the existence of publication bias. (**c**) Sensitivity plot.

The MMA of MT and TE on MMO in patients with TMD revealed a statistically significant moderate effect size [19,61,63] (SMD = 0.62; 95% CI 0.25 to 1.00; *p* < 0.001). Given that the I^2^ was <75% (I2 = 42%), and considering the results of Cochran’s Q statistical test (Q = 3.46 *p* = 0.18), the heterogeneity among the reviews was not considered significant. The shape of the funnel plot appeared to be symmetrical in the dominant model. The sensitivity exclusion analysis suggested that no review significantly affected the pooled SMD. Forest, funnel and sensitivity plots are shown in Figure 5. The % of overlap was 33.3% and the CCA = 0.08 (slight overlap). The overlapping of RCT is shown in Appendix, Table A5.

### 3.6. Effect of LLLT Interventions

Eight SR analyzed the effect of LLLT interventions on the management of TMD [18,52,53,54,59,62,64,67]. Seven of the SR evaluated the effects of LLLT on PI compared with placebo, and four SR found significant differences favoring LLLT for reducing PI at immediate post-intervention term (0 to 4 weeks) [52,53,62,67]. Five SR found no significant differences for reducing PI in the short term (2 to 4 weeks) [18,53,59,64,67]. Six studies evaluated the effect of LLLT on MMO compared with placebo, finding significant differences favoring LLLT in increasing MMO in the short term (0 to 4 weeks) [18,52,53,62,64,67] and 1 SR found no significant differences in MMO at immediate post-intervention [62].

In terms of the interventions analyzed in the SR, the main types of lasers used were GaAIAs, HeNe, GaAs, Nd: YAG, diode laser InGaAlP, infrared laser, AlGasAs, and gallium aluminum semiconductor LLL, with the control interventions being placebo, sham laser, or NSAIDs (piroxicam). Wavelengths ranged from 632 to 1064 nm, and dosage ranged from 0.3 to 144 J/cm^2^. Total intervention time varied from 10 s to 45 min, applied from 1 to 12 times per week, with a total number of sessions ranging from 1 to 24.

#### Quantitative Meta-Meta-Analysis of LLLT Intervention Results

**Figure 6:**
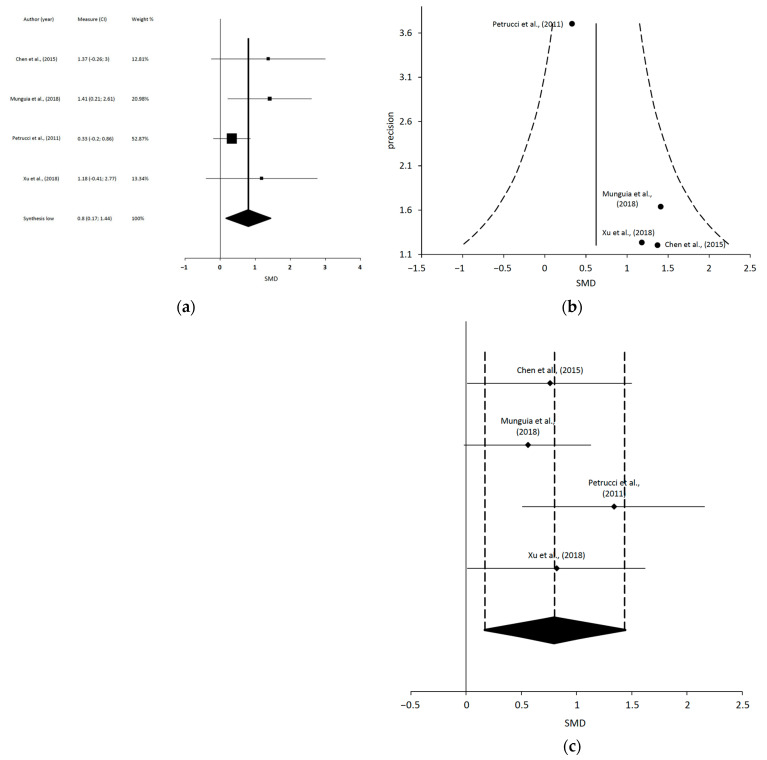
Synthesis forest and funnel plot of low-level laser therapy on pain intensity variable [18,62,64,67]. (**a**) The forest plot summarizes the results of included studies (standardized mean differences (SMD) and weight). The small squares represent the point estimate of the effect size and sample size. The lines on either side of the box represent a 95% confidence interval. (**b**) Funnel plot aims to assess the existence of publication bias. (**c**) Sensitivity plot.

Regarding the quantitative analysis, the MMA of LLLT on PI in patients with TMD revealed a statistically significant large effect size [18,62,64,67] (SMD = 0.8; 95% CI 1.44 to 0.17; *p* < 0.001). Given that the I^2^ was <75% (I2 = 27%), and considering the results of Cochran’s Q statistical test (Q = 4.12, *p* = 0.25), the heterogeneity among the reviews was not considered significant. The shape of the funnel plot appeared to be symmetrical in the dominant model. The sensitivity exclusion analysis suggested that the review of Muguia et al. influences the pooled SMD [62]. Forest, funnel and sensitivity plots are shown in Figure 6. The % of overlap was 41.6% and the CCA = 0.13 (slight overlap). The overlapping of RCT is shown in Appendix, Table A6.

**Figure 7:**
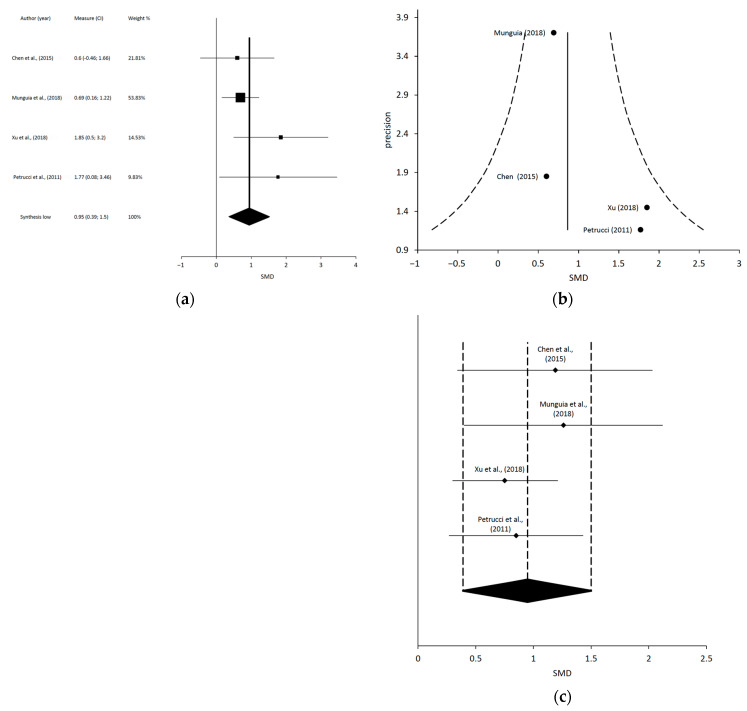
Synthesis forest and funnel plot of low-level laser therapy on maximum mouth opening variable [18,62,64,67]. (**a**) The forest plot summarizes the results of included studies (standardized mean differences (SMD) and weight). The small squares represent the point estimate of the effect size and sample size. The lines on either side of the box represent a 95% confidence interval. (**b**) Funnel plot aims to assess the existence of publication bias. (**c**) Sensitivity plot.

The MMA of LLLT on MMO in patients with TMD revealed a statistically significant large effect size (SMD = 0.95; 95% CI 1.5 to 0.39; *p* < 0.001). Given that the I^2^ was <75% (I^2^ = 21%) and considering the results of Cochran’s Q statistical test (Q = 3.81 *p* = 0.28), the heterogeneity among the reviews was not considered significant. The shape of the funnel plot appeared to be symmetrical in the dominant model. The sensitivity exclusion analysis suggested that no review significantly affected the pooled SMD. Forest and funnel plots are shown in Figure 7. The % of overlap was 35.7% and the CCA = 0.2 (slight overlap). The overlapping of RCT is shown in Appendix, Table A7.

### 3.7. Effect of Dry Needling Interventions

Three studies evaluated the effect of DN on the management of TMD [21,55,66]. Two SR found no significant differences between DN and sham DN on reducing PI in the short and intermediate term (2 weeks to 6 months), and 1 SR found better results for DN [21]. One SR found better results for DN than other wet needling interventions on reducing PI [66], and 1 SR found better results for DN than active placebo in increasing MMO in the intermediate term (2 weeks to 6 months) [55].

In terms of the interventions analyzed in the SR, DN consisted of the direct needling by a monofilament needle without any chemical agent injected directly into the masticatory or cervical muscles, and was compared with sham DN, wet needling, LLLT, and acupuncture. DN was compared with wet needling in 2 RSs [21,55], which were intramuscular injections with substances such as BTX, lidocaine, or granisetron, which were used in the masticatory muscles, and found the same positive effects for both interventions. Every SR studied the comparison of DN and sham DN, and contradictory results were found. Two SR found that DN was superior to sham DN [21,55]; however, 1 SR found no statistically significant difference in PI and MMO between both interventions [66]. In terms of the characteristics of the interventions applied, only 1 SR [66] described the total treatment time of the intervention, which was from 20 to 30 min, once a week for 1 to 5 weeks; and only 1 SR [55] described the total treatment sessions, which ranged from 1 to 4.

Despite 3 SR analyzed DN interventions an MMA could not be performed due to the inconsistencies in the results, the variability of the variables, and the interventions included.

### 3.8. Effect of Combined Physical Therapy Interventions

Seven SR analyzed the effect of combined PT interventions in patients with TMD [16,48,49,50,56,63,65]. When comparing combined PT interventions with placebo, no intervention, or usual care, four SR found significant differences in PI and MMO favoring PT interventions, mainly based on MT, TE, DN, LLLT, muscular awareness relaxation therapy, and self-management [48,49,50,63]. However, three SR found no significant differences [16,56,65]. Both the intervention and comparison groups analyzed in the SR were heterogeneous.

Interventions consisted of MT; TE (oral exercises, posture correction); occlusal splint therapy; electrotherapy modalities such as LLLT, transcutaneous electrical nerve stimulation (TENS), biofeedback, and photobiomodulation therapy; TEd (behavioral education, counselling); invasive interventions such as DN, acupuncture, oral injections, and surgeries; relaxation training; and counselling, all of which were applied in isolation or in combination.

Despite the number of SR that analyzed combined physical therapy interventions (7), an MMA could not be performed due to the heterogeneity of the interventions and variables and inconsistencies in the results.

### 3.9. Effect of Biofeedback Interventions

One SR of 10 RCTs [45] analyzed the effect of biofeedback training on the treatment of TMD, showing positive effects in reducing PI, reducing masticatory muscle activity, and decreasing frequency of sleep bruxism events, with a very low to moderate quality of evidence. Biofeedback training can be applied as visual biofeedback, vibratory biofeedback, audio biofeedback, or contingent electrical stimulation, and was compared with TENS treatment, occlusal splint therapy, and a control group. Total session time varied from 20 min to 8 h. The intervention lasted between 2 nights and 12 weeks, with a total number of sessions ranging from 2 to 42 sessions.

MMA could not be performed due to number of SR.

### 3.10. Grades of Evidence

The results of the grades of evidence of the MMA variables are shown in Table 5.

## 4. Discussion

The aim of this umbrella SR was to study, synthesize, and critically evaluate the current evidence on the effects of PT interventions in patients with TMD, specifically on pain and functional variables such as PI and MMO. We collected and synthesized evidence to assist with the decision-making process in the physical therapy and medical clinic setting. We specifically analyzed the presence of overlaps in the total included primary studies of the MMA, and we found a percentage of duplicities between 23.1% and 41.6%; moreover, the analysis of the CCA showed a slight overlap for all four MMA performed.

Various physical therapy interventions have been shown to be effective in improving pain and function in patients with TMD. Our MMA showed that combined interventions including MT and TE had statistically significant moderate effects on the improvement of pain, with a moderate grade of evidence; and on MMO, with a limited grade of evidence. Moreover, LLLT interventions had a statistically significant large effect on pain improvement, with a limited grade of evidence; and on MMO, with a moderate grade of evidence.

Two important symptoms of patients with TMD are pain and a reduction in MMO; thus, the use of therapeutic interventions that improve them appears critical. Interventions involving manual therapy and therapeutic exercise have been shown to be effective on improving these variables in patients with TMD; however, there is great diversity as to what the interventions consist of. From among the SR that analyzed these combined interventions and could be meta-meta-analyzed [15,19,57,60,61,63], the prescription of therapeutic exercise varied considerably, ranging from general exercises involving the whole body based on postural correction, to specific exercises of the TMJ and masticatory muscles, or even for the cervical spine. On the other hand, the use of manual therapy techniques was also diverse, including joint techniques such as mobilizations or manipulations of the cervical spine and/or TMJ, neural tissue mobilization techniques of the craniomandibular region, and soft tissue techniques such as compression and massage of painful muscles. In this sense, it is possible that the diversity of techniques found in the various SR is due to the diversity of the type of TMD. Different types of TMD are analyzed in the same SR, and therefore it could be that the use of certain techniques directed towards a specific tissue was not specifically analyzed, which would perhaps improve the effect of that specific intervention. This result highlights the importance of subclassifying patients with TMD so that we can make the decision of the choice of manual therapy techniques and exercise prescription towards the maximum effect for improving patients’ health. We should not forget that clinical reasoning based on the analysis of the symptoms’ source could be relevant for the choice of manual therapy technique, which is essentially how the DC/TMD subclassifies patients. There are two main general etiopathogenesis for the TMD which are myogenous and arthrogenous, they show with different clinical signs and symptoms, and treatment approach may be also different. However, many studies include different types of TMD or even mixed TMD in their samples. Another important topic to point out is the possible process of central sensitization that some patients could undergo when suffering chronic pain or in this case a chronic TMD, which could lead to a great pain expansion and overlapping of symptoms such as headache and fibromyalgia [68,69,70]. Additionally, psychological distress can contribute to the chronicity of the TMD, thus multidisciplinary management is necessary in these patients [68].

In future research, it will be essential to always classify and subclassify patients with TMD using clear criteria, such as according to the DC/TMD, as well as clearly define the chronicity of the TMD, so that the decision-making process and the impact of interventions could be analyzed, and their effectiveness in each subgroup can be compared.

In the studies included in the MMA, there was a lack of data regarding the intensity, dosage, and application frequency of manual therapy and exercise. A proper subclassification of TMD would also help in terms of finding the most appropriate therapy for each patient and the minimum effective dose. Another important aspect to note is the high frequency with which manual therapy is combined with therapeutic exercise in the scientific literature in the rehabilitation of patients with TMD. In this aspect, it is relevant to note that most research groups recommend the combination of both therapeutic tools for the management of these patients; their use in isolation does not appear to be the best choice.

Regarding usefulness, the combination of manual therapy and therapeutic exercise appears to be a useful intervention for the management of these patients regardless of the type of TMD; it has thus been proposed as a first-line treatment. Additionally, LLLT interventions have been shown to be effective for improving PI and MMO in patients with TMD, and the application appears to be more homogeneous. Among the SR that analyzed LLLT and could be meta-meta-analyzed [18,62,64,67], several types of LLLT were used. Although there was variability in the intensity and dose of application, the number of sessions was not greater than 20, with a weekly frequency of 1 to 12 sessions, applying the laser to the masticatory musculature and/or TMJ, with satisfactory results in terms of PI reduction and improvement in MMO in most of the SR. Unfortunately, due to the variability in the application of LLLT, MT, and TE, we cannot make a specific proposal of parameters for its application.

Given that the LLLT interventions’ follow-up time points were mostly in the short term, with a maximum follow-up of 3 months, we could not analyze the effects of these interventions on TMD. However, given that the follow-ups of the MT and TE interventions were conducted in the short, medium, and long terms, it could be interesting to investigate whether both interventions could be used in combination, using LLLT as a facilitating tool for the first phase of the whole rehabilitation process, with MT and ET as the main intervention. However, given that the combination of these interventions has not been analyzed in this umbrella SR, it is not possible to make a statement on this point.

### 4.1. Clinical Implications

As for the clinical application of this type of intervention, it was difficult to obtain sufficient information to generate concrete indications based on the data of this review; however, there are some indications that can be extracted to facilitate clinicians’ decision making.

First, this review suggests that conservative physiotherapy interventions using combined MT and TE or LLLT are the most studied interventions and are effective in the management of patients with TMD, and, given that these are conservative and low-cost interventions, they could be the best choice for the first line of treatment for these patients.

Second, subclassification of patients with TMD appears fundamental to improving decision making in terms of the choice of techniques and parameters in prescribing treatment, mainly considering the most likely source of symptoms.

Third, given that this review suggests that LLLT interventions achieve greater effects in the short term, but combined MT and TE achieve greater effects in the medium and long term on improving pain intensity and MMO, it is possible that the best intervention for these patients is a combination of both techniques, using LLLT in the initial phases (2–4 weeks) in combination with MT + TE, and maintaining the latter two for the medium to long term.

For the treatment planning and dosage, considering the data obtained from both interventions in this umbrella SR, we should consider sessions of between 30 and 60 min, with a frequency of 1 to 3 sessions per week, to be able to combine the interventions, dedicating less than 10 min to the LLLT application and 10 to 45 min to MT and TE, as well as being combined with home exercise.

Although the prescription of MT and TE appears simple to apply in the clinical field of physiotherapy, their effectiveness depends on the context in terms of the clinician and the patient, and always within a biobehavioral framework in which a multitude of social and psychological factors are involved and can enhance or worsen the results of the intervention.

### 4.2. Limitations

This umbrella SR has several limitations. The first is the lack of information on the interventions carried out in some RCTs in SR. Second, the review did not allow us to analyze the impact of educational and behavioral interventions, even though these interventions are used repeatedly among the reviews as part of the treatment, such as LLLT, MT, or TE, a fact that could influence the effect found in the application of the techniques. As a final limitation, given that we could not independently analyze acute and chronic TMD in the included SR, it was not possible to perform an analysis of the interventions’ effects in terms of chronicity in these different populations.

## 5. Conclusions

The present umbrella and mapping SR with MMA provides an overview of the effect of physical therapy interventions on PI and MMO variables in TMD. The results showed that the application of LLLT and combined MT and TE interventions have a positive effect on improving pain and functional variables in patients with TMD. The MMA showed the positive results of LLLT on PI and MMO, with a large effect size, and for combined MT and TE on PI and MMO, with a moderate effect size. The quality of evidence was limited for LLLT on PI and MT and TE on MMO, and moderate for LLLT on MMO and MT and TE on PI. The application of these interventions appears to be the first line in the treatment of TMD, and the combination of interventions should be considered in the clinical setting and in future studies.

## Figures and Tables

**Table 3:** Quality assessment scores and resultant reliability statistics (AMSTAR 2).

Study	1	2	3	4	5	6	7	8	9	10	11	12	13	14	15	16	Score	Overall Confidence
Al-Moraissi et al., 2020 [55]	2	2	0	1	2	2	0	2	2	0	2	2	2	2	2	2	25	Low
Al-Moraissi et al., 2020 [56]	2	2	0	1	2	2	0	2	2	0	2	2	0	2	2	2	23	Critically low
Alves et al., 2013 [57]	2	0	0	0	2	2	2	1	2	0	0	0	2	2	0	0	15	Critically low
Armijo-Olivo et al., 2016 [15]	2	2	0	1	2	2	0	2	2	0	2	2	2	2	2	2	25	Low
Brochado et al., 2019 [16]	0	0	0	0	2	2	0	0	2	0	0	0	0	2	0	0	8	Critically low
Calixtre et al., 2015 [17]	2	0	0	1	2	2	2	1	2	0	0	0	2	2	0	2	18	Critically low
Chen et al., 2015 [18]	2	0	0	0	2	2	0	1	0	0	2	0	0	2	2	2	15	Critically low
De Melo et al., 2020 [46]	0	0	0	0	2	2	2	2	2	0	0	0	2	2	0	2	16	Critically low
Dickerson et al., 2017 [19]	2	0	0	1	2	0	2	2	2	0	2	0	0	0	0	2	15	Critically low
Florjanski et al., 2019 [45]	0	0	0	0	2	2	0	1	0	0	0	0	0	0	0	2	7	Critically low
Fricton et al., 2009 [58]	0	0	2	0	2	0	0	1	2	0	2	0	0	2	0	2	13	Critically low
Herrera-Valencia et al., 2020 [20]	2	1	0	0	2	0	0	1	2	0	0	0	0	0	0	2	10	Critically low
Jing et al., 2021 [59]	2	2	0	0	2	2	0	1	2	0	2	2	2	2	2	2	23	Critically low
La Touche et al., 2020 [47]	2	1	0	1	2	2	0	2	2	0	0	0	0	2	0	2	16	Critically low
La Touche et al., 2020 [60]	2	1	0	1	2	0	0	1	2	0	2	2	2	2	2	0	19	Low
Machado et al., 2018 [21]	2	2	0	1	2	2	0	2	2	0	0	0	2	2	0	2	19	Critically low
Martins et al., 2016 [61]	2	2	0	0	2	0	0	1	2	0	2	2	2	2	2	2	21	Critically low
Mc Neely et al., 2006 [48]	2	0	0	2	2	0	2	1	2	0	0	0	2	2	0	0	15	Critically low
Medlicott et al., 2006 [49]	0	0	0	0	0	0	0	1	1	0	0	0	2	2	0	0	6	Critically low
Melis et al., 2019 [22]	0	0	0	2	2	0	0	2	2	0	0	0	2	2	0	2	14	Critically low
Melo et al., 2018 [51]	0	0	0	0	0	0	0	2	2	0	0	0	0	0	0	2	6	Critically low
Munguia et al., 2018 [62]	2	1	0	0	2	2	0	2	2	0	2	2	2	2	2	2	23	Critically low
Paço et al., 2016 [63]	2	0	0	0	2	2	0	1	2	0	2	2	2	2	2	2	21	Critically low
Petrucci et al., 2011 [64]	2	0	0	1	2	0	2	2	2	0	2	2	2	2	2	0	21	Low
Randhawa et al., 2016 [50]	2	2	0	0	2	2	0	2	2	0	0	0	2	2	0	0	16	Critically low
Tunér et al., 2019 [52]	2	1	0	0	2	1	2	1	1	0	0	0	0	2	0	2	14	Critically low
Lanas-Teran et al., 2019 [53]	2	1	0	0	2	0	0	0	2	0	0	0	0	2	0	2	11	Critically low
Van der Meer et al., 2020 [65]	2	2	0	0	2	2	2	2	2	0	2	2	2	2	2	2	26	Low
Vier et al., 2019 [66]	2	2	0	0	2	2	0	2	2	0	2	2	2	2	2	0	22	Critically low
Xu et al., 2018 [67]	2	0	0	0	2	2	0	2	1	0	2	2	2	2	2	2	21	Critically low
Zwiri et al., 2020 [54]	0	0	0	0	2	2	0	1	0	0	0	0	0	0	0	2	7	Critically low

Rating overall confidence in the results of the review. High: no or one non-critical weakness: Moderate: More than one non-critical weakness; low: one critical flaw with or without non-critical weaknesses; critically low: more than one critical flaw with or without non-critical weaknesses.

**Table 4:** Risk of bias assessment in systematic reviews through the ROBIS scale.

		Phase 2			Phase 3
Study	Study Elegibility Criteria	Identification and Selection of Studies	Data Collection and study Apraisal	Synthesis and Findings	Risk of Bias in the Review
Al-Moraissi et al., 2020 [55]	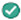	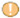	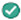	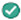	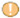
Al-Moraissi et al., 2020 [56]	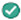	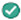	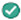	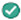	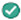
Alves et al., 2013 [57]	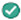	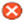	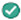	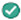	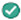
Armijo-Olivo et al., 2016 [15]	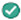	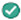	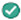	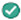	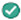
Brochado et al., 2019 [16]	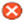	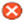	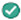	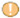	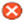
Calixtre et al., 2015 [17]	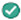	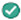	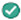	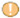	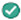
Chen et al., 2015 [18]	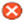	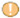	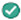	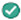	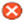
De Melo et al., 2020 [46]	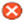	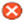	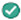	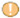	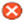
Dickerson et al., 2017 [19]	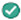	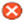	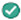	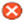	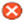
Florjanski et al., 2019 [45]	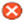	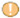	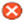	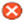	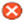
Fricton et al., 2009 [58]	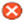	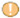	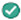	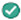	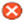
Herrera-Valencia et al., 2020 [20]	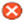	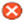	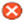	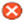	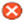
Jing et al., 2021 [59]	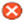	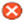	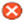	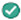	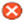
Lanas-Teran et al., 2019 [53]	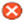	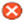	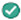	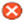	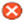
La Touche et al., 2020 [47]	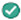	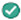	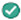	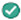	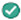
La Touche et al., 2020 [60]	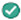	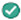	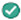	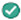	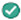
Machado et al., 2018 [21]	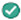	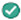	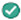	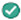	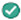
Martins et al., 2016 [61]	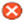	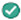	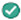	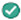	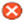
Mc Neely et al., 2006 [48]	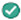	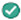	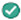	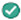	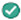
Medlicott et al., 2006 [49]	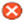	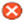	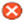	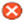	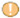
Melis et al., 2019 [22]	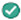	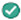	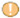	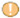	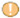
Melo et al., 2018 [51]	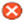	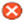	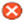	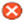	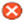
Munguia et al., 2018 [62]	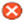	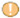	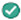	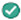	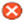
Paço et al., 2016 [63]	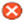	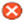	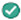	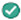	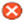
Petrucci et al., 2011 [64]	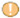	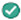	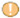	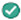	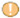
Randhawa et al., 2016 [50]	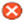	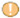	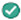	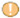	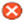
Tunér et al., 2019 [52]	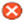	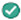	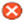	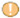	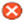
Van der Meer et al., 2020 [65]	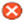	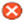	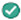	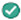	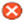
Vier et al., 2019 [66]	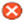	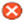	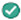	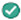	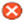
Xu et al., 2018 [67]	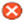	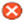	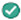	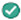	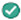
Zwiri et al., 2020 [54]	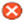	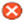	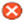	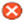	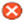

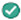
 = low risk; 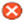 = high risk; 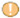 = unclear risk.

**Table 5:** Grades of evidence of meta-meta-analyzed variables (PAGAC).

Criteria						Effect	Evidence
Outcome. Intervention Type (Nº of Meta-Analyses)	Applicability	Generalizability	Risk of Bias or Study Limitations	Quantity and Consistency	Magnitude and Precision of Effect	SMD (95% CI)	
Pain intensity							
LLLT (4)	Strong	Moderate	Limited	Limited	Limited	0.8 (1.44 to 0.17)	Limited
MT + EX (4)	Strong	Moderate	Moderate	Limited	Moderate	0.51 (0.8 to 0.23)	Moderate
MMO							
LLLT (4)	Strong	Moderate	Limited	Limited	Moderate	0.9 (1.5 to 0.39)	Moderate
MT + EX (3)	Moderate	Moderate	Limited	Limited	Limited	0.62 (0.25 to 1.00)	Limited

Abbreviations: 95% CI: 95% confidence interval; LLLT: low-level laser therapy intervention; MT: manual therapy intervention; EX: therapeutic exercise intervention; MMO: maximum mouth opening; PPT: pressure pain threshold.

## Data Availability

All our data are published in the review, and can be found in the other paper we have reviewed.

## Appendix A

**Table A1:** Search strategies in each database.

Database	Search Strategy	Filters	Results (Date)
PubMed (1950 to 2021)	((“physical therapy”) OR (“manual therapy”) OR (“exercise”) OR (“dry needling”) OR (“soft tissue therapy”) OR (“soft tissue mobilization”) OR (“massage”) OR (“massage therapy”) OR (“therapeutic exercise”) OR (“education”) OR (“laser therapy”) OR (“oral myofunctional therapy”) OR (“relaxation”) OR (“relaxation techniques”) OR (“biofeedback”) OR (“cold”) OR (“thermotherapy”) OR (“cryotherapy”) OR (“heat”) OR (“TENS”) OR (“cold therapy”) OR (“heat therapy”) OR (“laser”) OR (“electrotherapy”) OR (“ultrasound therapy”) OR (“microwave”)) AND ((“temporomandibular joint”) OR (“temporomandibular disorders”) OR (“chronic temporomandibular disorders”) OR (“temporomandibular disorder”) OR (“myofascial masticatory pain”) OR (“masticatory muscle pain”) OR (“masticatory pain”) OR (“Headache attributed to TMD”) OR (“headache attributed to temporomandibular disorder”) OR (“Disc displacement with reduction”) OR (“temporomandibular degenerative joint disease”) OR (“temporomandibular subluxation”))	Systematic review Meta-analysis	105 (26 April 2021)
PEDro (1950 to 2021)	Abstract and Title: Temporomandibular Disorders Method: Systematic Review When Searching: Match all search terms (AND)	Not applicable	46 (26 April 2021)
SciELO (1997 to 2021)	((“physical therapy”) OR (“manual therapy”) OR (“exercise”) OR (“dry needling”) OR (acupuncture) OR (“soft tissue therapy”) OR (“soft tissue mobilization”) OR (“massage”) OR (“massage therapy”) OR (“therapeutic exercise”) OR (“education”) OR (“laser therapy”) OR (“oral myofunctional therapy”) OR (“relaxation”) OR (“relaxation techniques”) OR (biofeedback) OR (“cold”) OR (“thermotherapy”) OR (“cryotherapy”) OR (“heat”) OR (“TENS”) OR (“cold therapy”) OR (“heat therapy”) OR (“laser”) OR (“electrotherapy”) OR (“ultrasound therapy”) OR (“microwave”)) AND ((“temporomandibular joint”) OR (“temporomandibular disorders”) OR (“chronic temporomandibular disorders”) OR (“temporomandibular disorder”) OR (“myofascial masticatory pain”) OR (“masticatory muscle pain”) OR (“masticatory pain”) OR (“Headache attributed to TMD”) OR (“headache attributed to temporomandibular disorder”) OR (“Disc displacement with reduction”) OR (“temporomandibular degenerative joint disease”) OR (“temporomandibular subluxation”))	Review article	20 (26 April 2021)
LILACS (1998 to 2021)	((“physical therapy”) OR (“manual therapy”) OR (“exercise”) OR (“dry needling”) OR (acupuncture) OR (“soft tissue therapy”) OR (“soft tissue mobilization”) OR (“massage”) OR (“massage therapy”) OR (“therapeutic exercise”) OR (“education”) OR (“laser therapy”) OR (“oral myofunctional therapy”) OR (“relaxation”) OR (“relaxation techniques”) OR (biofeedback) OR (“cold”) OR (“thermotherapy”) OR (“cryotherapy”) OR (“heat”) OR (“TENS”) OR (“cold therapy”) OR (“heat therapy”) OR (“laser”) OR (“electrotherapy”) OR (“ultrasound therapy”) OR (“microwave”)) AND ((“temporomandibular joint”) OR (“temporomandibular disorders”) OR (“chronic temporomandibular disorders”) OR (“temporomandibular disorder”) OR (“myofascial masticatory pain”) OR (“masticatory muscle pain”) OR (“masticatory pain”) OR (“Headache attributed to TMD”) OR (“headache attributed to temporomandibular disorder”) OR (“Disc displacement with reduction”) OR (“temporomandibular degenerative joint disease”) OR (“temporomandibular subluxation”))	Systematic review	14 (26 April 2021)
EBSCOhost	“temporomandibular disorders” AND “physiotherapy”: 9 articles “temporomandibular disorders” AND “manual therapy” AND “systematic revision”: 4 articles “temporomandibular disorders” AND “laser” AND “systematic revision”: 4 articles “temporomandibular disorders” AND “tens” AND “systematic revison”: 0 articles “temporomandibular disorders” AND “exercise or physical activity” AND “systematic revision”: 15 articles “temporomandibular disorders” AND “relaxation” AND “SR”: 5 articles	Not applicable	37 (January 2021)
Google Scholar	With the exact sentence: “Temporomandibular disorders” With at least one of this: “physical therapy” “manual therapy” exercise “dry needling” “soft tissue therapy” “soft tissue mobilization” massage “massage therapy” Words shown: “in the tittle of the paper”	Not applicable	123 (January 2021)

**Table A2:** Reasons of exclusion of full-text screening process.

Database	Study	Reason for Exclusion
PUBMED	Buescher et al., 2007	Study Design
	Amorim et al., 2017	Population
	List et al., 2010	Study Design
	Christidis et al., 2019	Intervention
	Manfredini et al., 2015	Population
	Porporatti et al., 2019	Intervention
	Jung et al., 2011	Intervention
	Alajbeg et al., 2015	Intervention
	Wu et al., 2017	Intervention
	La Touche et al., 2010 (July-August)	Intervention
	Michiels et al., 2016	Population
	Cho et al., 2010	Intervention
	Türp et al., 2007	Intervention
	Al-Ani et al., 2005	Intervention
	Vos et al., 2013	Intervention
	Aggarwal et al., 2011	Intervention
	Türp et al., 2004	Intervention
	Tengrungsun et al., 2012	Population
	La Touche et al., 2010 (January)	Intervention
	Peixoto et al., 2021	Intervention
	Crider et al., 2005	Study Design
	Brosseau et al., 2007	Removed from Database
	Aggarwal et al., 2015	Removed from Database
	Ernst et al., 1999	Intervention
	Fernandes et al., 2017	Intervention
	Brantingham et al., 2013	Population
	Machado et al., 2019	Intervention
	Fertout et al., 2019	Study Design
	Stechman-Neto et al., 2016	Population
	Zhang et al., 2015	Intervention
	De Meurechy et al., 2019	Population
	Häggman-Henrikson et al., 2013	Population
	Tesch et al., 2021	Study Design
	Herpich et al., 2015	Study Design
	Melis et al., 2012	Study Design
	Maia et al., 2012	Study Design
	Shukla et al., 2016	Study Design
	Crider et al., 1999	Study Design
LILACS	Camara et al., 2014	Population
	Fragelli et al., 2013	Intervention
SciELO	Giovanna Pimentel et al., 2018	Intervention
	Fernanda Chiarion et al. 2018	Study Design
	Valenzuela-Chaigneau et al. 2018	Intervention
	Marcelo et al. 2017	Study Design
	Anieli da Costa et al. 2017	Study Design
	Andreia Valle de et al. 2021	Study Design
	Thânia Orlando et al. 2016	Intervention
	Moreira Moraes et al. 2015	Study Design
	Moreira Moraes et al. 2015 (Safar Giovanardi)	Study Design
	André Luís et al. 2015	Intervention
	Thaís C. et al. 2014	Intervention
	Machado et al. 2014	Study Design
	Nikita Gupta et al. 2013	Study Design
	Carvalho et al., 2012	Population
	Eduardo Grossmann et al., 2012	Study Design
	Assis et al., 2012	Study Design
	Maluf et al., 2008	Study Design
	Guinot-Moya et al., 2004	Study Design
	Conti et al., 2003	Study Design

**Table A3:** Funding information of included systematic reviews.

Article	Funding
Al-Moraissi et al., 2020 [55]	NA
Al-Moraissi et al., 2020 [56]	NA
Alves et al., 2013 [57]	NA
Armijo-Olivo et. al., 2016 [15]	Canadian Institutes of Health Research, the Alberta Innovates Health Solution, the STIHR Training Program of Knowledge Translation Canada and the Music and Motion Fellowship from the Faculty of Rehabilitation Medicine of the University of Alberta.
Brochado et al., 2019 [16]	NA
Calixtre et al., 2015 [17]	Brazilian National Council for Scientific and Technological Development
Chen et al., 2015 [18]	NA
de Melo et al., 2020 [46]	NA
Dickerson et al., 2017 [19]	NA
Florjanski et al., 2019 [45]	NA
Fricton et al., 2009 [58]	National Institute of Dental and Craniofacial Research’s TMJ Implant Registry and Repository
Herrera-Valencia et al., 2020 [20]	Department of Physiotherapy, Faculty of Health Sciences, University of Málaga (Spain)
Jing et al., 2020 [59]	National Natural Science Foundation of China
La Touche et al., 2020 [47]	NA
La Touche et al., 2020 [60]	NA
Machado et al., 2018 [21]	NA
Martins et al., 2016 [61]	NA
McNeely et al., 2006 [48]	NA
Medicott et al., 2006 [49]	NA
Melis et al., 2019 [22]	NA
Melo et al., 2018 [51]	NA
Munguia et al., 2018 [62]	NA
Paco et al., 2016 [63]	Institute of Research and Advanced Training in Health Sciences and Technologies
Petrucci et al., 2011 [64]	NA
Randhawa et al., 2016 [50]	Canadian Research Chairs programs
Turner et al., 2019 [52]	NA
Lanas-Teran et al., 2019 [53]	NA
Van der Meer et al., 2020 [65]	NA
Vier et al., 2019 [66]	NA
Xu et al., 2018 [67]	National Natural Science Foundation of China, Shaanxi Province Natural Science Basic Research Foundation of China, and key discipline foundation of Xi’an Medical University
Zwiri et al., 2020 [54]	University Sains Malaysia

NA, not available.

**Table A4:** Overlap of clinical trials of the MMA of combined manual therapy and therapeutic exercise interventions on pain intensity.

	Included Systematic Reviews and Meta-Analyses			
Clinical Trials	Alves et al., 2013 [57]	Armijo-Olivo et al., 2016 [15]	Paço et al., 2016 [63]	La Touche et al., 2020 [60]
Minakuchi et al., 2001	x	x		
Schiffman et al., 2007	x	x		
Carmeli et al., 2001		x	x	
Haketa et al., 2010		x		
Ismail et al., 2007		x		
Craane et al., 2012 a			x	
Cranee et al., 2012 b			x	
Kalamir et al., 2012			x	
Kalamir et al., 2013			x	
Tuncer et al., 2013			x	
Corum et al., 2018				x
La Touche et al., 2013				x
Calixtre et al., 2018				x

**Table A5:** Overlap of clinical trials of the MMA of combined manual therapy and therapeutic exercise interventions on mouth opening.

	Included Systematic Reviews and Meta-Analyses		
Clinical Trials	Martins et al., 2016 [61]	Paço et al., 2016 [63]	Dickerson et al., 2017 [19]
Taylor et al., 1994	x		
Ismali et al., 2007	x		
Cuccia et al., 2009	x		
Von Piekartz et al., 2010	x		
Tuncer et al., 2013	x	x	
Carmeli et al., 2001		x	
Craane et al., 2012a		x	x
Craane et al., 2012b		x	
Kalamir et al., 2012		x	x
Kalamir et al., 2013		x	
Fernández-Carnero et al., 2010		x	
Felicio et al., 2010			

**Table A6:** Overlap of clinical trials of the MMA of LLLT interventions on pain intensity.

	Included Systematic Reviews and Meta-Analyses			
Clinical Trials	Chen et al., 2015 [18]	Mungia et al., 2018 [62]	Petrucci et al., 2011 [64]	Xu et al., 2018 [67]
Carrasco et al., 2008	x		x	x
De Cunha et al., 2008	x		x	
Demirkol et al., 2015	x	x		x
Emshoff et al., 2008	x		x	
Ferreira et al., 2013	x			
Kulekcioglu et al., 2003	x		x	
Marini et al., 2010	x			x
Sattayut et al., 2012	x			
Venancio et al., 2005	x			x
Carrasco et al., 2009		x		x
Cetiner et al., 2006		x		
De Moraes Maia et al., 2014		x		
Venezian et al., 2010		x		
Conti et al., 1997			x	
De Abreu venancio et al., 2005			x	
Carrasco et al., 2009b				x
Carrasco et al., 2009c				x
De Carli et al., 2013				x
Mazzetto et al., 2017				x
Wang et al., 2001				x

**Table A7:** Overlap of clinical trials of the MMA of LLLT interventions on mouth opening.

	Included Systematic Reviews and Meta-Analyses			
Clinical Trials	Chen et al., 2015 [18]	Mungia et al., 2018 [62]	Petrucci et al., 2011 [64]	Xu et al., 2018 [67]
Ahrari et al., 2014	x			x
Da Silva et al., 2012	x			x
Kulekgioglu et al., 2003	x		x	x
Marini et al., 2010	x			x
Venancio et al., 2005	x			x
Carrasco et al., 2009		x		
Cetiner et al., 2006		x		
De Moraes Maia et al., 2014		x		
Demirkol et al., 2015		x		
Venezian et al., 2010		x		
De Abreu venancio et al., 2005			x	
Röhling et al., 2011				x
Wang et al., 2011				x
Conti et al., 1997				x
